# Development of an application programming interface to automate downloading and processing of precision livestock data

**DOI:** 10.1093/tas/txae092

**Published:** 2024-06-07

**Authors:** Jameson R Brennan, Ira L. Parsons, Meredith Harrison, Hector M Menendez

**Affiliations:** Department of Animal Science, South Dakota State University, Rapid City, SD 57703, USA; Department of Animal Science, South Dakota State University, Rapid City, SD 57703, USA; C-Lock Inc., Rapid City, SD 57703, USA; Department of Animal Science, South Dakota State University, Rapid City, SD 57703, USA

**Keywords:** cattle production, data science, near real-time data acquisition, precision technology

## Abstract

Advancements in technology have ushered in a new era of sensor-based measurement and management of livestock production systems. These sensor-based technologies have the ability to automatically monitor feeding, growth, and enteric emissions for individual animals across confined and extensive production systems. One challenge with sensor-based technologies is the large amount of data generated, which can be difficult to access, process, visualize, and monitor information in real time to ensure equipment is working properly and animals are utilizing it correctly. A solution to this problem is the development of application programming interfaces (**APIs**) to automate downloading, visualizing, and summarizing datasets generated from precision livestock technology (**PLT**). For this methods paper, we develop three APIs and accompanying processes for rapid data acquisition, visualization, systems tracking, and summary statistics for three technologies (SmartScale, SmartFeed, and GreenFeed) manufactured by C-Lock Inc (Rapid City, SD). Program R markdown documents and example datasets are provided to facilitate greater adoption of these techniques and to further advance PLT. The methodology presented successfully downloaded data from the cloud and generated a series of visualizations to conduct systems checks, animal usage rates, and calculate summary statistics. These tools will be essential for further adoption of precision technology. There is huge potential to further leverage APIs to incorporate a wide range of datasets such as weather data, animal locations, and sensor data to facilitate decision-making on time scales relevant to researchers and livestock managers.

## Teaser

As precision livestock technology advances, processes are needed to automate data access and systems checks. The use of application programming interfaces will facilitate technology adoption and integration of real-time data into decision-making processes.

## INTRODUCTION

The advancement of precision livestock technology (**PLT**) has ushered in a new era of sensor-based data collection to measure and monitor animal production (Menendez et al., 2022). These technologies offer many opportunities to better understand animal nutrition and growth models, animal response to environmental stress (Fuchs et al., 2019), supplement intake (Durst et al., 2022), feed efficiency (Siberski-Cooper and Koltes, 2022), and animal health (Schillings et al., 2021). It is estimated that PLT could result in a $20 billion increase in agricultural production (Baker et al., 2017). The most widely used technologies for livestock include real-time animal tracking, in pasture weighing systems, precision feeding systems, and measuring enteric emissions. Though there are a lot of promises for PLT for research and on farm applications, one of the main challenges associated with these technologies is the ability to readily access and process data generated from these sensors in time scales relevant for management decisions.

Though animal scientists are well trained in research and statistical design of experiments, they are not trained in data science principles and may lack the necessary skills to tackle many of the big data challenges associated with precision technology (Jacobs et al., 2022). These skills include downloading large datasets that are generated daily, creating data pipelines to automate processing and cleaning of precision data, generating visualizations to assess quality issues, identifying individual animal non-adoption with precision technology, and incorporating data into animal nutrition or machine learning models (Brennan et al., 2023a; Dagel, 2023; Jacobs et al., 2023; Parsons et al., 2023). The time required to clean, process, and manage large and complex data can require up to 80% of the work effort on a project (Light et al., 2014). Though we have a tremendous amount of information at our disposal using PLT, for it to be useful at relevant management and research scales it needs to be accessed in real time to ensure successful implementation of precision technology. Furthermore, as different research groups utilize the same technology, disparate data processing tools are often developed to handle large datasets leading to a lack of standardization for data processing, duplication of research efforts across sites, and disparity in methodologies for cleaning and processing data.

Application programming interfaces (APIs) are software or code that allows two applications to communicate with each other. APIs are frequently used to access and share data hosted on a server in the cloud within or across organizations. For the purpose of this paper, APIs that are being discussed refer to code that has been developed in an open-source statistical software platform (e.g., R or Python) that serves the function of importing sensor data from PLTs into statistical software platforms for further cleaning, visualization, analysis, and reporting of data. APIs have been developed to access a wide range of data relevant for decision support tools for agriculture including monitoring land use and crop production at continental scales (Xiong et al., 2017; Zhang et al., 2020) and incorporating weather and climate data to inform pasture management for grazing livestock (Thomasa et al. 2019). There is huge potential to incorporate near real-time datasets via APIs into decision support tools for livestock production and research; however, documentation and open-source tutorials are often lacking (Brennan et al., 2023a). Without the construction of reliable open-source PLT-API code, it is likely that the development and application of near real-time decision tools that enhance livestock production and sustainability will remain difficult.

The overall goal of this paper was to develop open-source tools that overcome the largest barrier to PLT implementation and use, which are rapid and repeatable PLT data access, system tracking, and descriptive statistics. Therefore, the objectives of this paper were to 1) develop APIs to automate data access and basic data processing and visualization in Program R; 2) demonstrate daily system tracking processes that monitor whether PLT is functioning and collecting the expected quality of experimental data; and 3) demonstrate common post-trial summary statistics. These three specific objectives will be demonstrated for three unique PLTs that include individual animal precision feeding (kg DMI/d), weighing (kg/d), and enteric emissions (g/d of CH_4_, CO_2_, H_2_, and O_2_ consumption).

## METHODS

### API Development

We developed three technology separate APIs, system tracking processes, and descriptive statistics in Program R for three technologies manufactured by C-Lock Inc. The API developed utilizes the package “rvest” (Wickham, 2021) with a RESTful API architecture to automate downloading and processing of all three technologies (SmartScale, SmartFeed, and GreenFeed). Within each technology, users must enter login credentials, equipment ID, and desired dates of data request. Tutorials for the SmartScale (Supplementary Material), SmartFeed (Supplementary Material), and GreenFeed (Supplementary Material) APIs were created as R Markdown documents to help users run the API and associated code; all code is also available on a GitHub webpage (https://github.com/sdsu-cottonwood-precision-ranch/C_Lock_API). In addition, technology-specific example datasets are provided to enable users to run actual data through the C-Lock interface with a “guest login” for the SmartScale and SmartFeed technologies to facilitate greater learning and adoption of these tools.

Since data were provided directly by C-Lock from an archived database, there was no prior animal care and use protocol approved by an institutional animal care and use committee. The SmartScale, SmartFeed, and GreenFeed systems collect real-time data to generate daily records on animal weights, intake, and emissions, respectively. All monitoring systems are equipped with a radio frequency identification (**RFID**) reader to record individual animal RFID tags. The systems upload data each hour using internet connection through Wi-Fi or local cellular networks. Data available on the API are dependent on the system type. For the SmartScale units, the data are typically processed and available once per hour. For the SmartFeed units, the data are processed and available once per hour and is typically available 1 h after data are uploaded from the system. For the GreenFeed units, the data are typically processed once per day. Delays in data being uploaded via cellular or Wi-Fi connection or a large number of systems being processed simultaneously can occasionally cause lags in data availability on the API of over one day. Of note, data available in near real time on the API are considered processed preliminary data by the manufacturer, which are data that have been converted into meaningful values through an algorithm. Processed finalized data are typically provided by the manufacturer after the conclusion of the study and may take 1-4 weeks for final verification.

### Precision Weighing

The SmartScale is an automated weighing system that is used to measure individual animal body weight (**BW**) in confinement and grazing systems. The platform scale system is manufactured to be positioned in front of a water trough, so that an animal must step their front feet on the scale to drink water. C-Lock converts front-quarter, partial body weight (**pBW**) to full BW using a conversion factor of 1.76. The conversion factor has been internally derived from C-Lock verification data. Within the API call, users can specify full BW data using the C-Lock conversion factor or pBW values and apply a customized conversion factor post downloading. Similar conversion factors between pBW and full BW measurements have been observed across multiple classes of beef cattle using SmartScale units (Brennan et al., 2023b). C-Lock processed data files for SmartScale data include daily weights summarized by individual animal providing a generic report. Daily weights are calculated as the average of all the sub-daily weight “events” that are considered valid. Daily weight estimates and individual event data are both able to be accessed via the API, where individual events are defined as each unique interaction of an animal with a PLT. The SmartScale data used in this analysis included records for one scale for 126 days and daily weight records on seventy-four individual animals. The SmartScale precludes multiple animals from accessing the unit simultaneously, this resulted in 3,779 individual daily weight records for the example dataset.

### Precision Feeding

SmartFeed is a system for measuring individual animal intake and feeding behavior. The feed bin is positioned on two load cells and the weight of the feed bin is measured at 1-s intervals. C-Lock algorithms use data from each of the load cells to calculate “Feed Mass” and the “Baseline Mass” for each SmartFeed bin. The baseline mass is used for calculations of individual animal intake by calculating disappearance (e.g., baseline mass at time 1 − baseline mass at time 2). With the SmartFeed system animals can be given ad libitum access to feed or be restricted through an automated headgate system. Processed data for SmartFeed include daily as-fed intake summarized by animal and individual animal feeding events (i.e., bunk visits). SmartFeed data used in the analysis were collected in parallel with the SmartScale data using five SmartFeed units, resulting in 3,775 individual daily intake records.

### Enteric Emissions

The GreenFeed is a portable, free-access respiration enclosure that measures individual animal CO_2_, CH_4_, and H_2_ gas production and O_2_ consumption using mass fluxes. The technology is described in detail by Hristov et al. (2015). Briefly, GreenFeed dispenses a pelleted bait feed with user-configured settings to encourage animal visitation. The system uses a high airflow rate to sample animal breath, and machine sensors measure real-time airflow, gas concentrations, environmental factors, and animal head proximity. C-Lock developed GreenFeed data algorithms identify “good observations” data which are made available on the server for downloading via the API. For this paper, “good observations” are defined as data that have undergone an initial postprocessing step which uses a proprietary algorithm to filter bad records. Data removal criteria includes flux calculations when the animal’s head is too far from the manifold, instances when multiple animals are reaching into the unit, instances when the wind is blowing directly into the system, and records where the CO_2_ and CH_4_ standard deviations are within acceptable ranges. The processing algorithm continuously monitors ambient background concentrations and adjusts for airflow and other dimensional correction factors. Additionally, the processing algorithm calculates the mass of feed delivered by the GreenFeed for each animal based on the total number of machine “cup drops” and the average mass of a cup drop (e.g., 32 g). Similar to the SmartScale and SmartFeed technologies, summarized data are available as daily individual animal and herd averages. The “Count” data denote the number of animal events that were used in the daily animal and herd averages. GreenFeed summarized data were also accessible in the “events” format. Processed events data columns returned by the API are provided in the GreenFeed markdown document (Supplementary Material). For the GreenFeed markdown tutorial, GreenFeed data were downloaded and the number of daily observations and individual observations of “good visit” data are plotted, as well as average methane and carbon dioxide emissions per animal. In addition, the hour of the day was derived from timestamp data to plot methane emissions by hour of day to visualize diurnal patterns in emissions data. GreenFeed data used in the analysis were collected independently from the SmartScale and SmartFeed data. GreenFeed data used to generate the plots are not publicly available; however, users can adjust the login credentials to download and view datasets generated from the GreenFeed system.

## RESULTS AND DISCUSSION

We were successful in developing three unique APIs for precision weighing, feeding, and enteric emission PLT using Program R (Supplementary Material). We demonstrate examples of data cleaning, system tracking, and general summary statistics for each PLT below and describe potential benefits to their use.

### Precision Weighing

**Figure 1.**
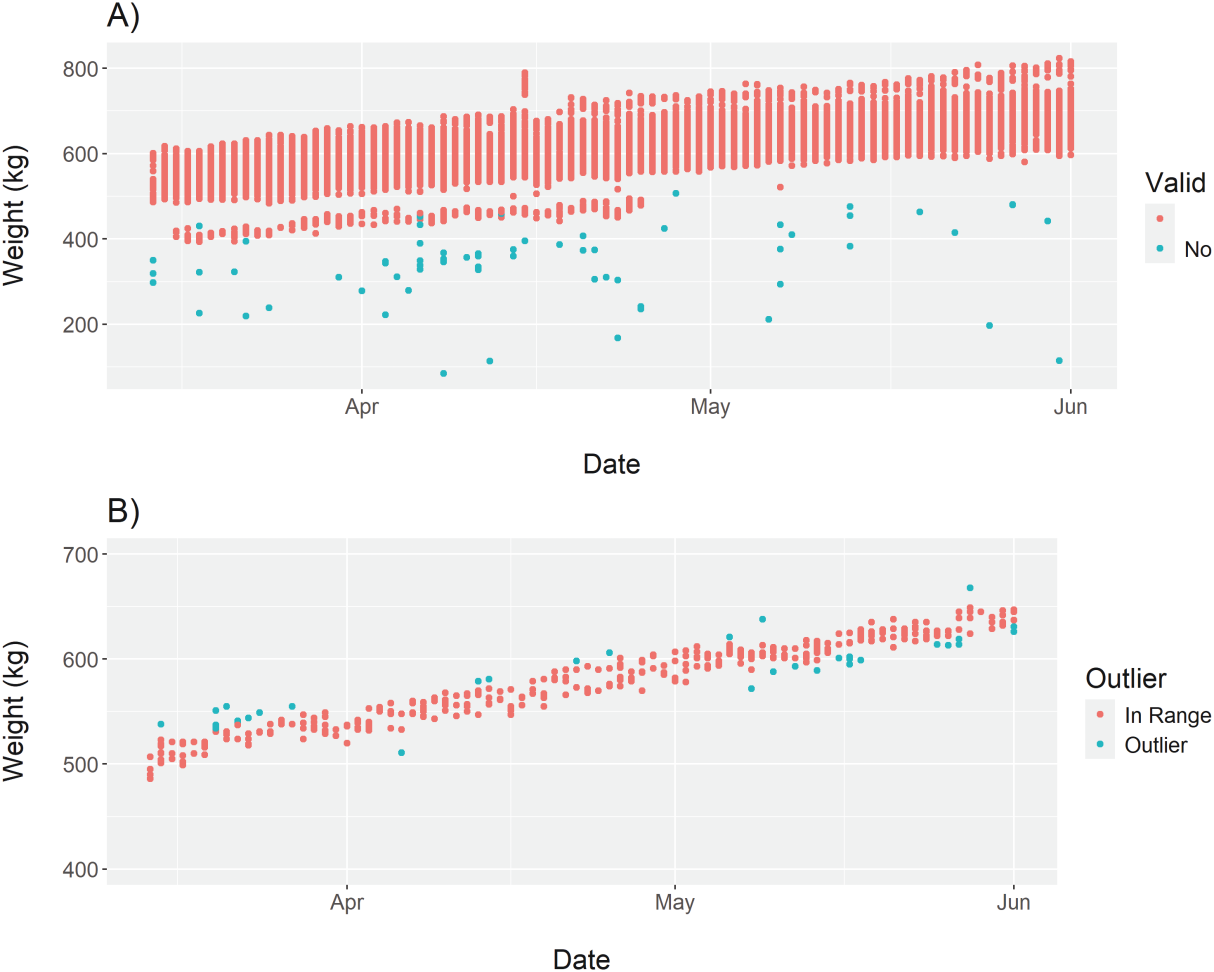
A time series plot of animal weight by time generated by SmartScales (C-Lock Inc.). (A) shows the entire herd data with points flagged as invalid by the C-Lock system. The plot in (B) shows an individual animal’s weight data through time with outlier data flagged using robust regression.

The result of the SmartScale API example successfully downloaded 11,204 individual animal visit observations with dates ranging from March 14, 2023 to June 1, 2023 into the R environment. Further data processing steps within the example removed unnecessary columns and converted the 14-digit RFID to the last 6 digits for ease of reading. In addition, data processing steps cleaned bad data flagged within the C-Lock system as well as applied robust regression equations to remove potentially outlier weight data points from the dataset (Figure 1; Parsons et al., 2023). A total of 9,053 individual animal visit observations remained following the data-cleaning process. Systems tracking plots were generated to show the number of unique animal visits per scale per day and the number of observations by day. These plots can be used to quickly identify sudden changes in animal visits which could indicate either equipment malfunction or issues with water access for animals. Finally, the SmartScale code was used to create basic summary statistics of average daily gain (**ADG**) and starting weight for each individual animal. To accomplish this, a linear regression equation was fitted for each individual animal with animal weight as the dependent variable and the day of trial as the independent variable. The resulting analysis generated a new data set with animal RFID, starting weight (intercept of the linear model), and ADG (slope of the linear model). Processes developed could be used to track animal weight gains in real-time to ensure animals are meeting performance targets or potentially identify changes in animal growth that might indicate changes in environmental conditions or animal health.

### Precision Feeding

**Figure 2.**
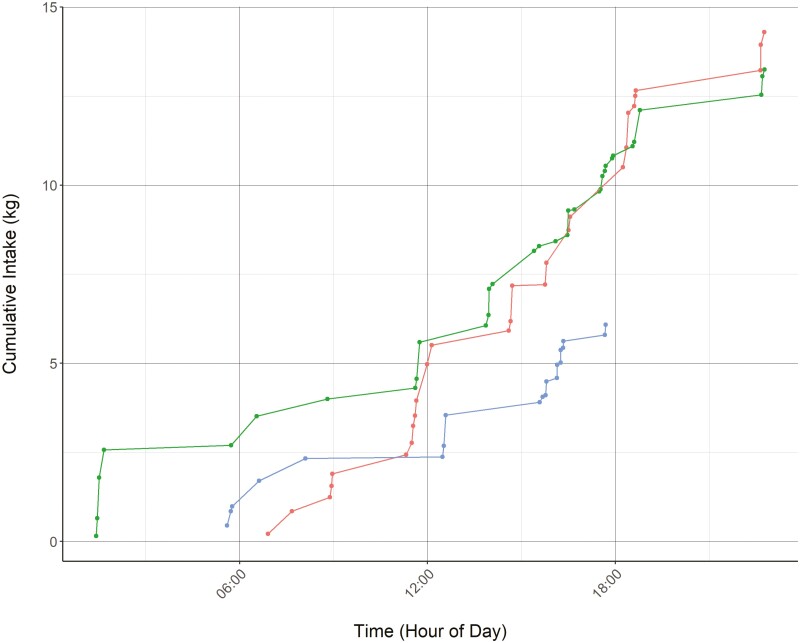
Plot of feed intake for three individual animals measured using the SmartFeed system (C-Lock, Inc.). The plot shows cumulative intake over the course of a day and can be used to assess animal feed consumption per visit or to assess if feed limits have been met.

SmartFeeds provide insight into feeding behavior, such as visit duration, frequency, eating rate, and feed intake. Using the API to access this data, both during and after the completion of a study, providing a number of advantages, including informing animal management, machine functionality, and ensuring the repeatability of results. The result of the SmartFeed API example successfully downloaded 152,484 individual feeding observations. Data processing steps included in the markdown tutorial deleted the unnecessary columns, created a new column called “Date” that converts the start time to a date-only value, and converted the 14-digit RFID number to only the last 6 digits to simplify identifying unique animals. Additional steps include plotting and removing bad data flagged as not valid by the C-Lock system. System checks are performed on the SmartFeed system by plotting the bunk visit frequency by date. These plots can be used to rapidly assess whether animals are utilizing the feed bunks at regular intervals and quickly identify sudden changes that may indicate either equipment malfunctions or feed issues. Lastly, basic summary statistics are provided to calculate daily intake (kg) for individual animals and daily cumulative intake (kg) for individual animals (Figure 2). This information can be used to determine feeding behavior patterns of animals, analyze between different treatment groups, or determine whether cumulative intake amounts are met when animals are fed a restricted diet.

### Precision Enteric Emissions

**Figure 3.**
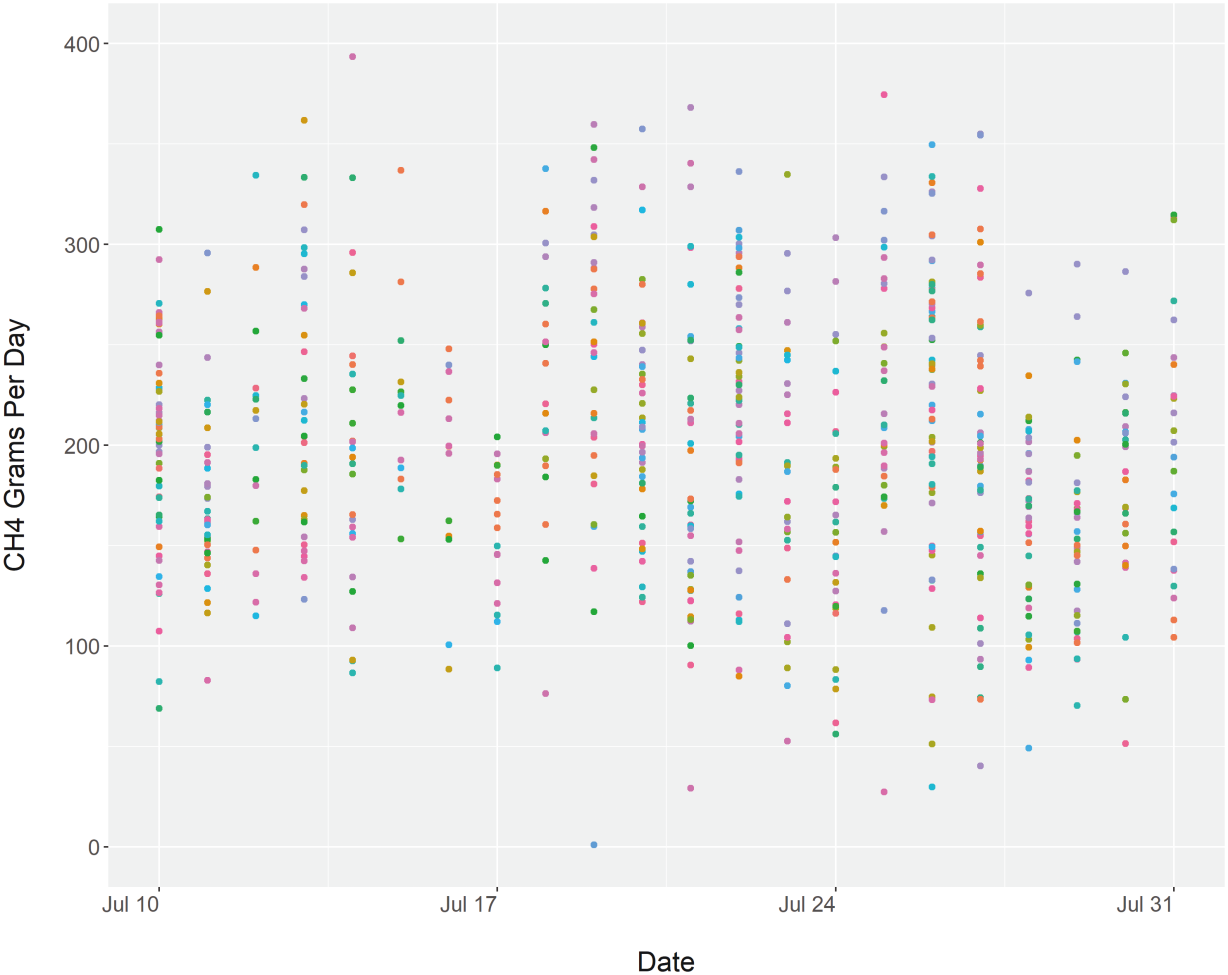
The amount of average daily methane (CH_4_) emitted per animal per day for a single GreenFeed unit over a 20-day period. Individual colors represent individual animals. Quick visualization plots like the amount of methane produced per day can be used to assess if measurements are within expected ranges for enteric emissions.

The GreenFeed API provides a rapid method of authenticating credentials and parsing data into a data frame resulting in column names for specific variables (e.g., “CH4GramsPerDay”). The data requested by the GreenFeed API Markdown document undergo a preprocessing step by C-Lock to only report “good observations” (Arthur et al., 2017). Utilizing the API to access GreenFeed data not only increases the ease of downloading data and creating repeatable analysis, but also facilitates the daily observation of animal visitation behavior and emissions output. For example, systems tracking plots for the total number of daily visits per GreenFeed and the number of “good observations” can be used to rapidly track changes in animal use patterns and serve as an indication should an intervention be needed (Figure 3). System tracking for the GreenFeed is important for several reasons. Firstly, the user is able to quickly determine whether the machine is actively collecting data. Secondly, the evaluation of the number of good observations can be compared to experimental requirements such as a minimum sample size. If the expected number of daily GreenFeed visits is not being met, the user can further troubleshoot GreenFeed function (e.g., hardware, software, and cellular data transmission) and physical access of cattle to the machine (e.g., empty feed hopper, mud, and alley width). Lastly, the user can quickly evaluate animal technology adoption (i.e., user/non-user) and use rates. Example summary statistics include the visualization of individual enteric emissions measurements per day (Figure 3). This is important to quickly check if measurements are within the expected or desired range for each gas type (e.g., 143.6 to 372.7 or 309 g/d CH_4_ in beef animals (McGinn et al., 2021; Min et al., 2022).

Such data can be extrapolated to broader applications of GreenFeed to evaluate the number of individual visits per period. Dressler et al. (2023) reported that a minimum number of 38 spot samples is required to measure enteric methane emissions for an individual grazing animal. Similarly, Manafiazar et al. (2016) indicated that a minimum of 20 spot samples over a 7- or 14-day sampling period produced measurements with low variability in gas emissions, or 50 to 100 spot samples over a 4-week period depending on the research question (Renand and Maupetit, 2016). Thus, the user may further add to the API to assess desired research criteria (i.e., number of individual visits or mean daily individual enteric values) and place tolerance limits to send alerts for reporting. These real-time reports provide opportunities for rapid intervention to help users better manage their equipment, animals, and experimental timelines.

## CONCLUSIONS

The development of APIs allows data transfer in a uniform and secure manner, creating a pathway to access PLT data quickly and easily. Common analytical languages (e.g., R and Python) utilize APIs to access precision livestock data and perform user-defined data processing and analytical algorithms. These are central to developing and testing methodologies to inform real-time decision-making tools. Further, it creates a programmatic method to download and process data, create visual tools and summary tables, and directly write manuscripts and reports in a traceable manner that ensures repeatability of analyses.

Methods developed in this manuscript can facilitate the near real-time implementation of analytical processes, a necessary step toward utilizing precision technology to inform day-to-day management and research decisions. Monitoring animal growth and performance is a critical attribute of many research trials. Continuous tracking of ADG as a cohort and individual performance metrics provide insight into potential management strategies by succinctly presenting the data to inform management decisions. This data can be further leveraged to create statistical process control charts which may provide a means of monitoring and reporting individual animal variance relative to metabolically critical events or to identify the onset of Bovine Respiratory Disease, in high-risk calves (Kayser et al., 2019). There is huge potential to further leverage APIs to incorporate a wide range of datasets such as weather data, animal locations, and sensor data into reports or data dashboards to inform livestock management decisions (Nyamuryekung’e et al., 2023); the limitation being how quickly data, either raw or processed, are available to the end user. Though the focus of this paper is to present three separate API tutorials for downloading and processing data, the open-source code provided allows users the opportunity to manipulate the source code from multiple technologies to generate additional metrics, such as calculating feed-to-gain ratios based on SmartScale and SmartFeed data collected concurrently.

## Supplementary Material

txae092_suppl_Supplementary_Material
