# Supplementary material for: Development of an application programming interface to automate downloading and processing of precision livestock data
Source: Transl Anim Sci. 2024 Jun 7;8:txae092. doi: 10.1093/tas/txae092 (PMC11209544; doi:10.1093/tas/txae092)
Supplement: txae092_suppl_Supplementary_Material [file txae092_suppl_supplementary_material.zip › GreenFeed_API.pdf]

Downloading GreenFeed data via an API in R

Authored by Dr. Jameson Brennan, Department of Animal Science, South Dakota State University

The objectives of this markdown document is to automate the download and visualization of Greenfeed data provided by C-Lock. Users will need to provide their own login credentials to run the code. This markdown can be used to quickly visualize usage of Greenfeed machines and preliminary data.

This is an R Markdown document. Markdown is a simple formatting syntax for authoring HTML, PDF, and MS Word documents. For more details on using R Markdown see http://rmarkdown.rstudio.com. When you click the **Knit** button a document will be generated that includes both content as well as the output of any embedded R code chunks within the document. The example below will print a statement and run a quick computation.

```
knitr::opts_chunk$set(echo = TRUE)
library(ggplot2)
```

R Markdown

This is an R Markdown document. Markdown is a simple formatting syntax for authoring HTML, PDF, and MS Word documents. For more details on using R Markdown see http://rmarkdown.rstudio.com.

When you click the **Knit** button a document will be generated that includes both content as well as the output of any embedded R code chunks within the document.

Enter your variables

The first thing you need to do is to enter your variables for the code to run. In the quotations below you will need to change the username, password, FID (or greenfeed ID), and the start and end times for the data you want to download.

Two things to note are 1) you can enter multiple greenfeeds assocated with an account and 2) the start time and end time must be in the same format. The chunk below sets the end time to the computer time for the most recent downloads.

```
#change to login user name
USER <- "User_Name"

#Change to login password
PASS <- 'Password'

#Change to greenfeed id or id's. The second line can be uncommented out for multiple greenfeeds
FID  <- "541"
#FID  <- "541,539,535,534"

#enter the start date (ST) and end date (ET) for the data you want to download
ST   <- "2023-06-06"
ET   <-  Sys.Date()
```

API Code

This code chunk will pull the data from the cloud based on the specifications of what was provided above.

```
# Spaces must be replaced with %20
```

```r
library(httr) # Must first install  httr with:   install.packages("httr")
library(stringr)
# First Authenticate to receive token:
req <- POST("https://portal.c-lockinc.com/api/login", body=list(user=USER, pass=PASS))
stop_for_status(req)
TOK <- trimws(content(req))


#URL="https://portal.c-lockinc.com/api/getemissions?d=visits&fids=297,298&st=2023-06-06%2000:00:00&et=2


# Now get data using the login token
URL=paste0("https://portal.c-lockinc.com/api/getemissions?d=visits&fids=", FID, "&st=", ST, "&et=", ET,
req <- POST(URL, body=list(token=TOK))
stop_for_status(req)

a <- content(req)

#Split the lines
perline <- str_split(a, "\\n")[[1]]

#Split the commas into a dataframe, while getting rid of the "Parameters" line and the headers line
df <- do.call("rbind", str_split(perline[3:length(perline)], ","))
df=as.data.frame(df)

colnames(df)=c('FeederID','AnimalName','RFID','StartTime','EndTime','GoodDataDuration',
                   'CO2GramsPerDay','CH4GramsPerDay','O2GramsPerDay','H2GramsPerDay','H2SGramsPerDay',
                   'AirflowLitersPerSec','AirflowCf','WindSpeedMetersPerSec','WindDirDeg','WindCf',
                   'WasInterrupted','InterruptingTags','TempPipeDegreesCelsius','IsPreliminary','RunTim
```

Quick Visualization Plots

This next section is used to generate a series of quick plots to summarize visits and data from the greenfeeds to check usage rates.

```r
#convert start time to date
df$Date=as.Date(df$StartTime)

df$RFID=stringr:: str_sub(df$RFID,-6,-1)

#get a count on the number of good observations by each greenfeed by day
library(dplyr)
daily_good_data=df %>% count(FeederID, Date, sort = TRUE)

daily_good_data=na.omit(daily_good_data)
ggplot(daily_good_data,aes(x=Date,y=n))+
  geom_bar(stat = 'identity')+
  facet_wrap(~FeederID)+
  ggtitle('Number of good observations by GF by day')+
  ylab('Number of daily observations')
```

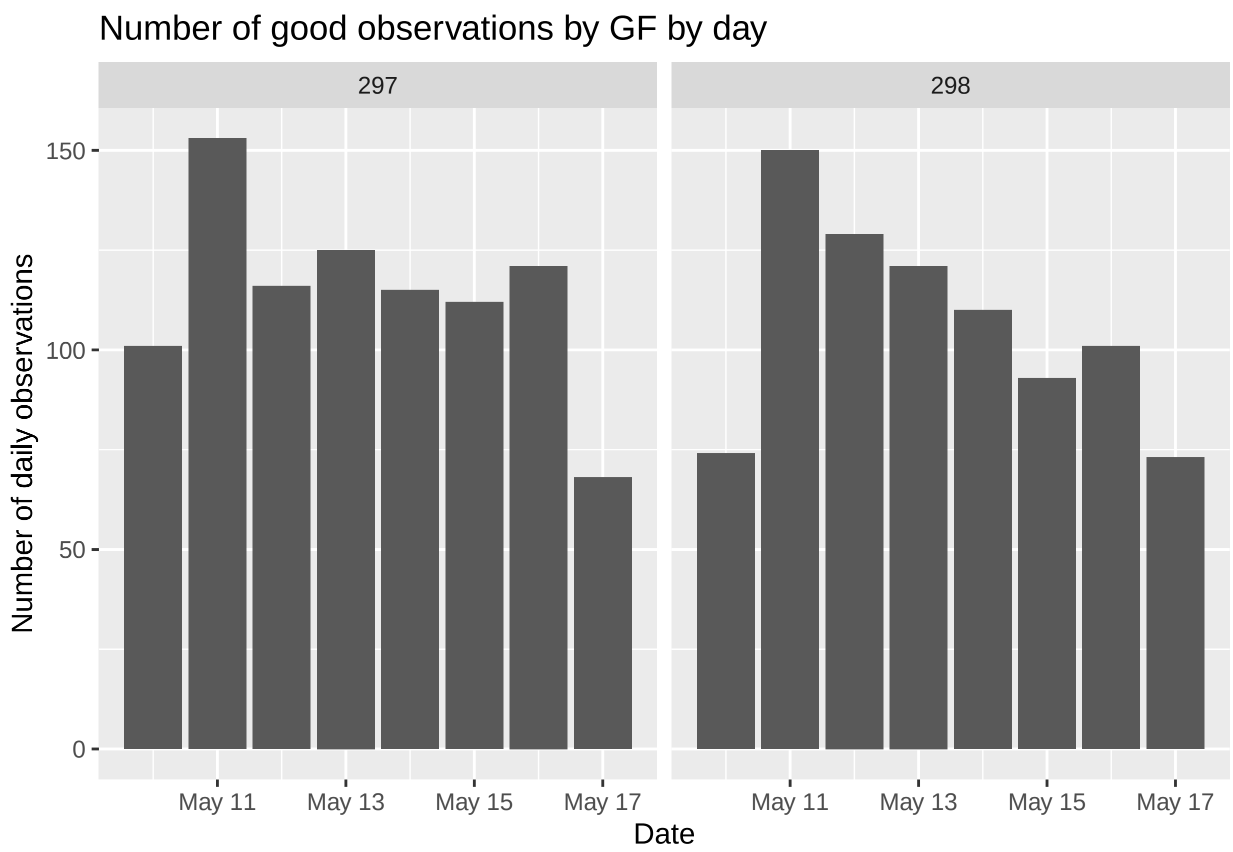


```
#get the number of unique animals visiting by day
rfid_day=df %>%                                    # Applying group_by & summarise
  group_by(Date,FeederID) %>%
  summarise(count = n_distinct(RFID))
```

```
## `summarise()` has grouped output by 'Date'. You can override using the
## `.groups` argument.
```

```
rfid_day=na.omit(rfid_day)

ggplot(rfid_day,aes(x=Date,y=count))+
  geom_bar(stat = 'identity')+
  facet_wrap(~FeederID)+
  ggtitle('Number of Unique animals per day')
```

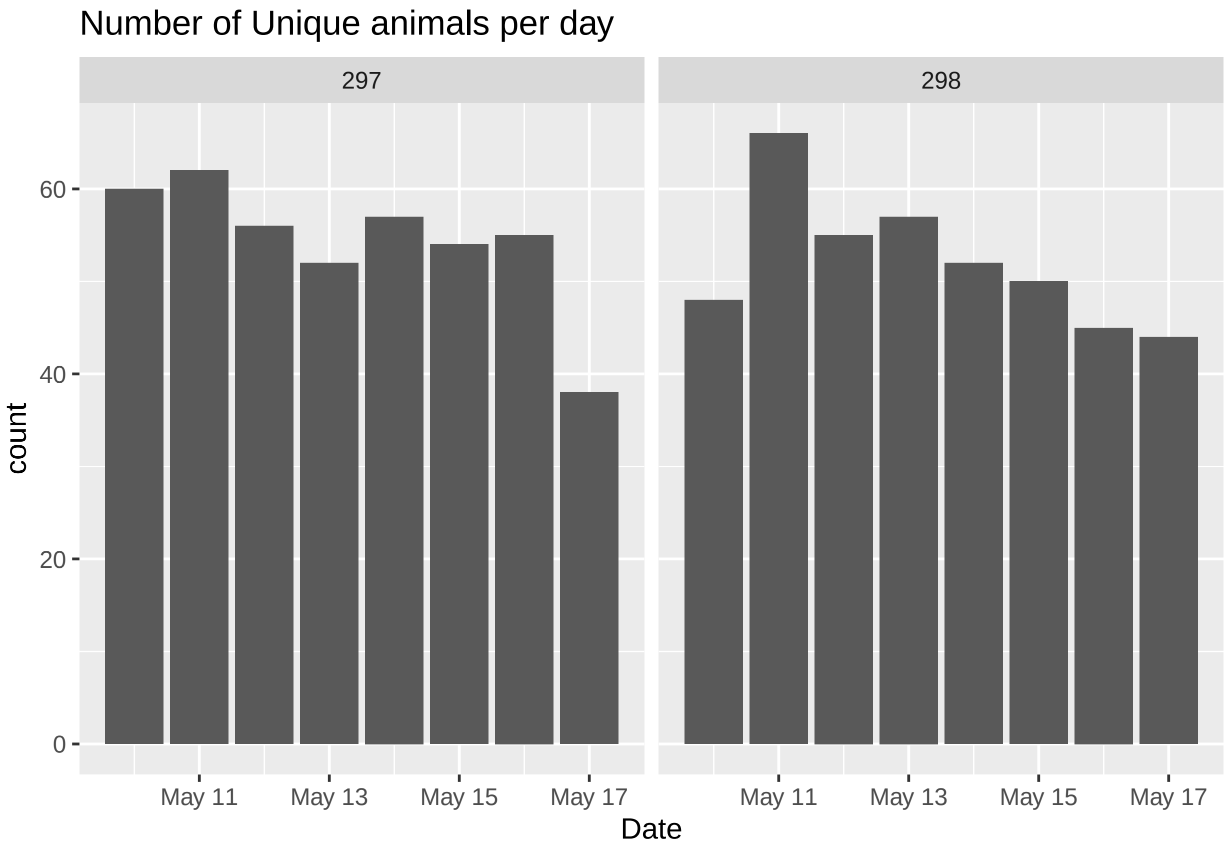


```
df=na.omit(df)

df$CH4GramsPerDay=as.numeric(df$CH4GramsPerDay)

ggplot(df,aes(y=CH4GramsPerDay,x=Date,color=RFID ))+
  geom_point()+
  ylim(0,300)+
  ggtitle('Grams CH4 per day, colors individual animals ')+
  theme(legend.position = "none")
```

```
## Warning: Removed 6 rows containing missing values or values outside the scale range
## (`geom_point()`).
```

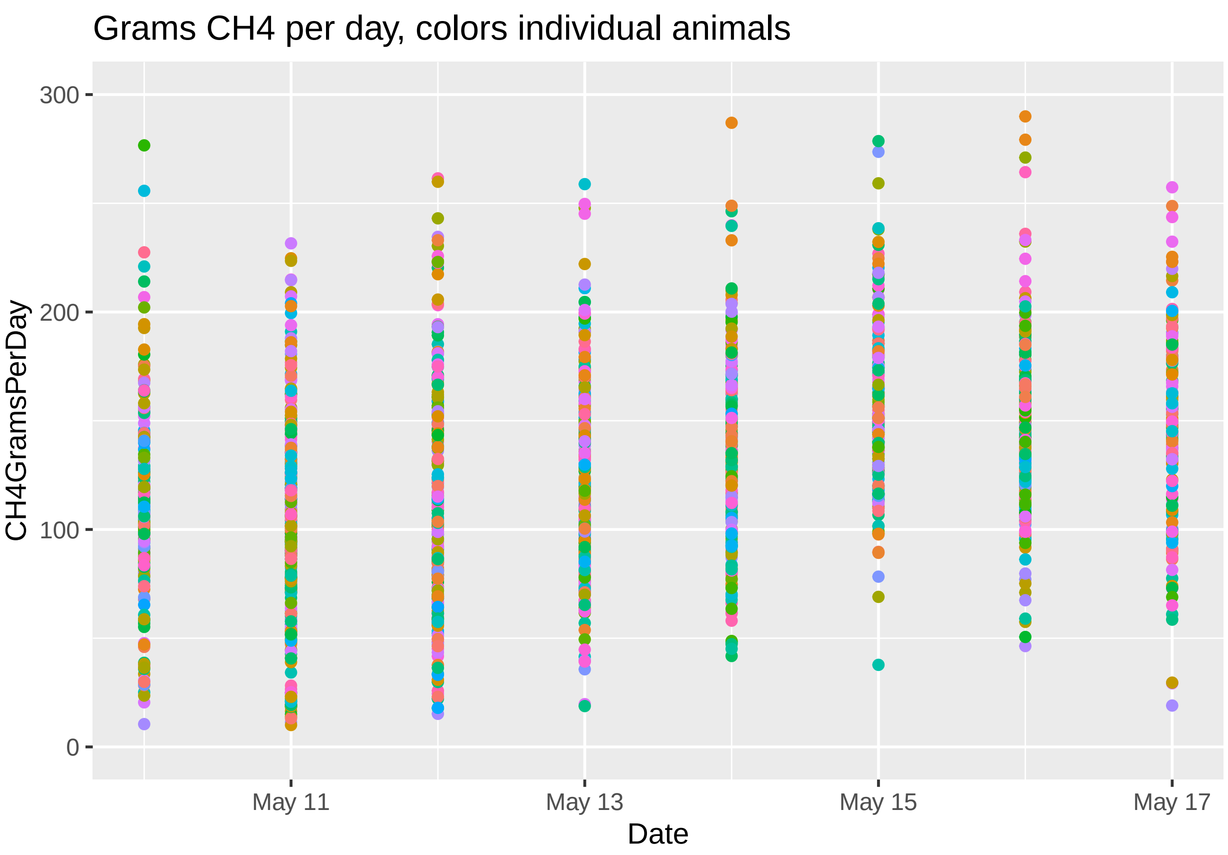


```r
df$CO2GramsPerDay=as.numeric(df$CO2GramsPerDay)

ggplot(df,aes(y=CO2GramsPerDay,x=Date,color=RFID ))+
  geom_point()+

  ggtitle('Grams CO2 per day, colors individual animals ')+
  theme(legend.position = "none")
```

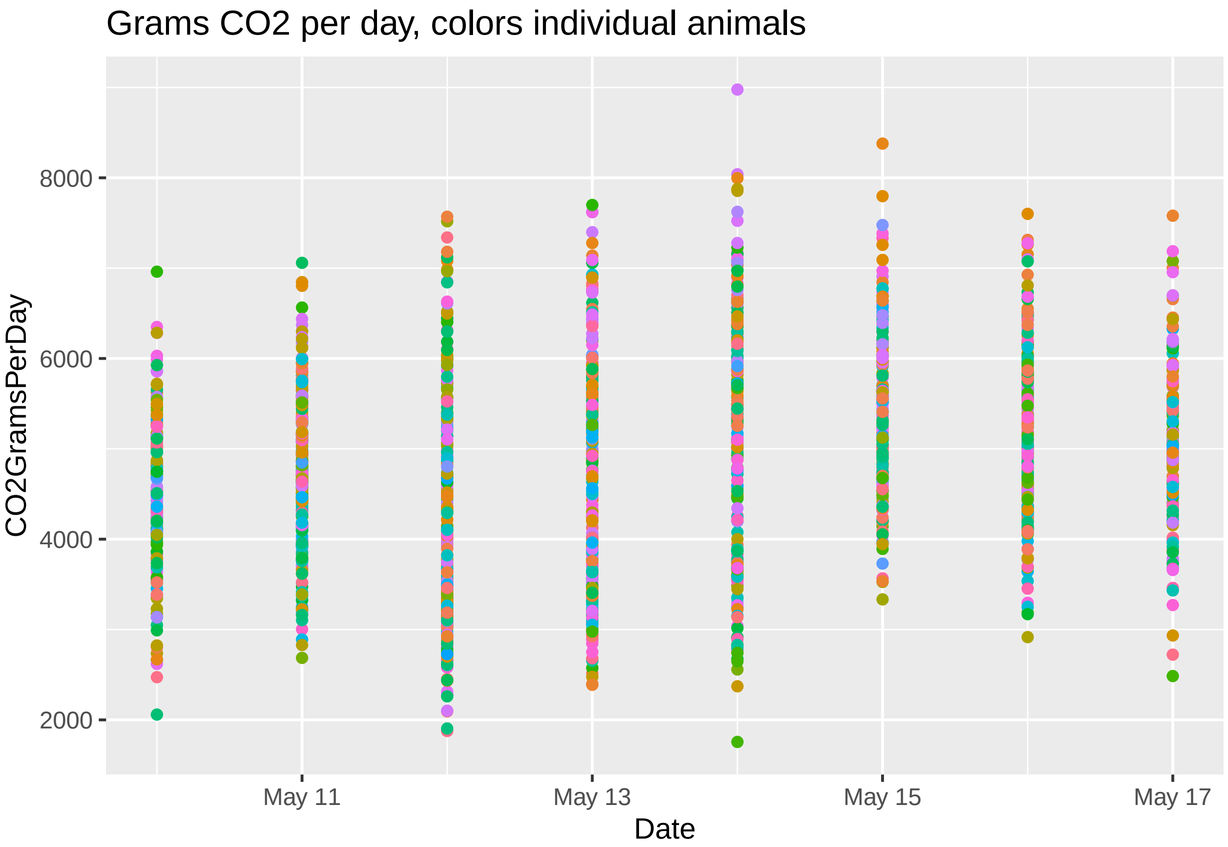
Grams CO2 per day, colors individual animals
CO2GramsPerDay
8000
6000
4000
2000
May 11
May 13
May 15
May 17
Date
