# Supplementary material for: Development of an application programming interface to automate downloading and processing of precision livestock data
Source: Transl Anim Sci. 2024 Jun 7;8:txae092. doi: 10.1093/tas/txae092 (PMC11209544; doi:10.1093/tas/txae092)
Supplement: txae092_suppl_Supplementary_Material [file txae092_suppl_supplementary_material.zip › SmartFeederTutorial.pdf]

C-Lock SmartFeeder Tutorial

Downloading and Processing C-Lock SmartFeeder Data in R

Developed by Jameson Brennan, Ira Parsons, and Hector Menendez

Department of Animal Science, South Dakota State University The objectives of this hands on tutorial are to introduce workshop participants to methods for streamlining SmartFeeder data processing tasks in R.

This is an R Markdown document. Markdown is a simple formatting syntax for authoring HTML, PDF, and MS Word documents. For more details on using R Markdown see http://rmarkdown.rstudio.com. When you click the **Knit** button a document will be generated that includes both content as well as the output of any embedded R code chunks within the document. The example below will print a statement and run a quick computation.

Download API data

Import libraries

Our first step to processing the data is to import the libraries we will use to run our analysis. Each library contains a set of functions which can be used to process data. For example, the function mean() would sum the values in a column and divide by the number of observations in the column. This code will look to see if the necessary packages are installed on your computer and if not install and load them.

```
##if there is an error and a package or dependency needs to be updated un-comment the
#code below and replace 'rvest' with package
#remove.packages('rvest')
#install.packages('rvest')

#Needed packages
  list.of.packages <- c("rvest",'httr','RCurl','tidyverse','data.table','lubridate',
                        'knitr','markdown','fasttime','MASS')
  new.packages <- list.of.packages[!(list.of.packages %in%
                                      installed.packages()[,"Package"])]
  if(length(new.packages)) install.packages(new.packages)
library(rvest)
library(httr)
library(RCurl)
library(tidyverse)
library(data.table)
library(lubridate)
library(knitr)
library(rmarkdown)
library(fasttime)
library(MASS)
```

Enter login credentials

Next, we need to create character variables specifying the login credentials for the application interface. In the quotations, enter your username and password.

```
#change to login user name
USERNAME <- 'demo_user'

#Change to login password
PASSWORD <- 'greenfeed'
```

Enter report paramters

The next step is to enter the parameters for our data query.In the quotations below you will need to change the FID (or SmartFeeder ID), and the start and end times for the data you want to download.

Two things to note are 1) you can enter multiple pieces of equipment assocated with an account and 2) the start time and end time must be in the same format. We have entered an end time since our data collection for this project is complete. However, you can change this parameter to current computer time to download the most recent dataset.

```
## Equipment assignments -----
equipIDs = c('10818,10819,10820,10821,10822')

intake.type = 'visits' # Type of data requested

## Date start and end -----
date.start <- as.POSIXct('2023-03-14', format = '%Y-%m-%d', tz = 'UTC')
date.end   <- as.Date('2023-07-18',format = '%Y-%m-%d', tz = 'UTC')
```

API code

This code chunk will pull the data from the cloud based on the specifications of what was provided above.

```
# Login ----
# First Authenticate to receive token:
req <- POST("https://portal.c-lockinc.com/api/login", body=list(user=USERNAME, pass=PASSWORD))
stop_for_status(req)
TOK <- trimws(content(req))

# Set up URL ----
url = paste0('https://portal.c-lockinc.com/api/',
             'getintake?d=',intake.type, # Specify vists or daily average
             '&fids=',equipIDs, # Specify feeder ids
             '&st=',date.start, # Specify start date
             '&et=',date.end # Specify end date
)

# Download data ----
req = POST(url,body = list(token=TOK))
stop_for_status(req)

# Parse data ----
ct = str_split(content(req, as = 'text'), '\\n')[[1]]
d.sfeed = data.table(do.call('rbind', str_split(ct[3:length(ct)],",")))
colnames(d.sfeed) = c(str_split(ct[2],',')[[1]])
```

```
# Convert RFID to 15 digit number
d.sfeed$RFID = str_sub(d.sfeed$RFID,-6,-1)

# Save raw feeding events dataset
# fwrite(dt, file = 'Data/211-SmartFeed.csv')
```

System tracking

Viewing and cleaning SmartFeeder data

This is the raw dataset downloaded from the SmartFeeder API.The code chunk will print the first five rows of data in table format.

```
knitr::kable(head(d.sfeed))
```

FeederID	RFID	StartTime	EndTime	Duration	StartMassKG	EndMassKG	IntakeKG	FeedType	WarningCode	WarningMsg	SSFTray
10818	605896	2023-03-14 18:00:29	2023-03-14 18:02:42	133	121.708	121.222	0.486	1	0		0
10818	605892	2023-03-14 18:08:33	2023-03-14 18:21:39	786	121.247	118.65	2.597	1	0		0
10818	605896	2023-03-14 18:21:39	2023-03-14 18:28:08	389	118.65	118.199	0.45	1	0		0
10818		2023-03-14 18:28:08	2023-03-14 18:58:44	1836	118.199	114.214	3.986	1	3	Unallocated feed.	0
10818	679559	2023-03-14 18:58:44	2023-03-14 19:05:44	420	114.214	113.067	1.146	1	0		0
10818	679617	2023-03-14 19:05:44	2023-03-14 19:09:48	244	113.067	111.937	1.131	1	0		0

We can see that the 'SSFTray' column is likely not necessary and can be removed. In addition, we will create a new column called 'Date' that converts the start time to a Date only value. Lastly we will convert the 14 digit RFID number to only the last 6 digits to simplify identifying unique animals.

```
#Remove unnecessary columns
d.sfeed$SSFTray=NULL

#convert date time to date value to look at daily visits
d.sfeed$Date=as.Date(d.sfeed$StartTime)
```

Quick Visualization Plots

One of the first steps to processing and cleaning data is to plot it. This code chunk will get the number of daily visits for each feeder and plot it by day.

The smartfeeder data has a column named 'Valid' that flags potentially bad data in the system based on quantiles. The following plots show the bad data labeled as Valid = No for the entire dataset and for an individual animal.

```r
#plot animal weight by date
# ggplot(d.sfeed,aes(x=Date,y=IntakeKG,color=WarningMsg))+
#   geom_point()+
#   theme(legend.position = 'none')

d.sfeed %>%
  dplyr::filter(RFID=='605896' & WarningMsg != "") %>%
  group_by(Date,WarningMsg) %>%
  mutate(Count = n()) %>%
  ggplot(aes(x=Date, y = Count, fill=WarningMsg))+
  geom_col()+
  theme_classic()+
  theme(panel.grid.major = element_line(color = 'black', linewidth = 0.1),
        panel.grid.minor = element_line(color = 'grey', linewidth = 0.05),
        plot.caption = element_text(hjust = 0),
        legend.position = "bottom")
```

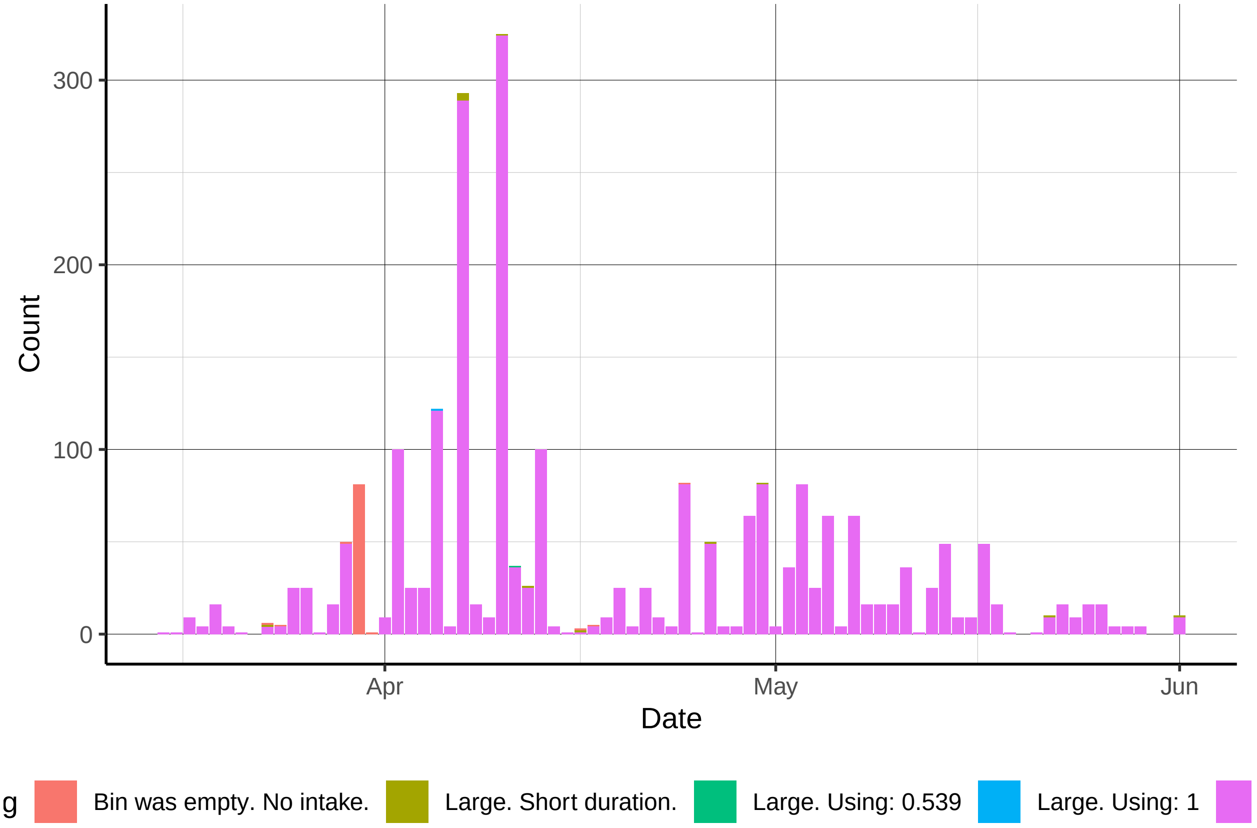

This next session shows a few examples of quickly calculating animal behavior to summarize daily animal visit behavior to quickly observe utilization rates. These plots are helpful to ensure equipment is functioning as expected, and that animals are using the equipment as desired.

```r
# FeedingBehavior -----
d.fbdaily = d.sfeed[, .(BVfreq = .N,
                         BVdur.tot = sum(as.numeric(Duration), na.rm = T),
                         BVdur.u = mean(as.numeric(Duration), na.rm = T),
                         BVdur.sd = sd(as.numeric(Duration), na.rm = T),
                         IntakeKG.tot = sum(as.numeric(IntakeKG), na.rm = T),
                         ERkg.min = (sum(as.numeric(IntakeKG), na.rm = T)/(sum(as.numeric(Duration), na
```

```
                 by = list(RFID,Date)]

# Calculate cumulative intake
setorder(d.fbdaily, cols = 'RFID','Date')
d.fbdaily[, cumDMI := cumsum(IntakeKG.tot), by = 'RFID']
```

Now, we have applied processing algorithms to calculate animal feeding behaviors, including feed intake. Next, we can quickly plot some of these behaviors.

```
p.bvfreq = d.fbdaily %>%
  dplyr::filter(RFID %in% c('680417',
                            '679459',
                            '679471')) %>%
  ggplot(aes(as.Date(Date), y = BVfreq))+
  geom_point(aes(color = RFID))+
  geom_smooth(aes(color = RFID), se = F)+
  geom_smooth(se = F)+
  labs(x = 'Date',
       y = 'Bunk visit frequency, n per day',
       title = 'Bunk visit frequency')+
  theme_classic()+
  theme(panel.grid.major = element_line(color = 'black', linewidth = 0.1),
        panel.grid.minor = element_line(color = 'grey', linewidth = 0.05),
        axis.text.x = element_text(angle = 45, hjust = 1),
        plot.caption = element_text(hjust = 0),
        legend.position = "bottom")
p.bvfreq
```

```
## `geom_smooth()` using method = 'loess' and formula = 'y ~ x'
## `geom_smooth()` using method = 'loess' and formula = 'y ~ x'
```

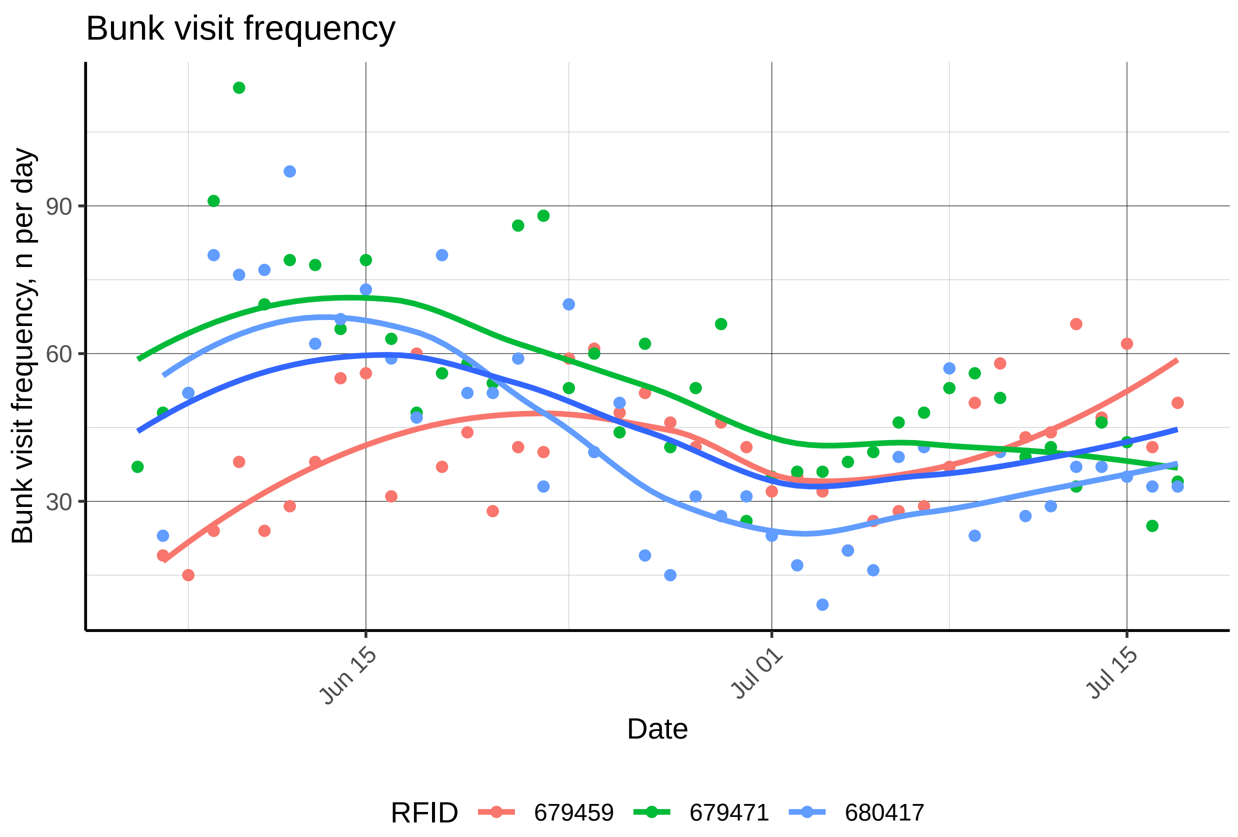

This next chunk of code will remove the observations that are not valid and replot the data.

```
#Remove not valid points
d.sfeed=subset(d.sfeed, WarningMsg == "")
# FeedingBehavior -----
d.fbdaily = d.sfeed[, .(BVfreq = .N,
                         BVdur.tot = sum(as.numeric(Duration), na.rm = T),
                         BVdur.u = mean(as.numeric(Duration), na.rm = T),
                         BVdur.sd = sd(as.numeric(Duration), na.rm = T),
                         IntakeKG.tot = sum(as.numeric(IntakeKG), na.rm = T),
                         ERkg.min = (sum(as.numeric(IntakeKG), na.rm = T)/(sum(as.numeric(Duration), na
                 by = list(RFID,Date)]

# Calculate cumulative intake
setorder(d.fbdaily, cols = 'RFID','Date')
d.fbdaily[, cumDMI := cumsum(IntakeKG.tot), by = 'RFID']

#plot animal weight by date
p.intakedaily = d.fbdaily %>%
  dplyr::filter(RFID %in% c('680417',
                            '679459',
                            '679471')) %>%
  ggplot(aes(x = as.Date(Date), y = IntakeKG.tot, color = RFID))+
  geom_point()+
  geom_smooth(se = F)+
  labs(x = 'Date',
       y = 'Daily intake, kg per day',
       title = 'Intake')+
```

```
  theme_classic()+
  theme(panel.grid.major = element_line(color = 'black', linewidth = 0.1),
        panel.grid.minor = element_line(color = 'grey', linewidth = 0.05),
        axis.text.x = element_text(angle = 45, hjust = 1),
        plot.caption = element_text(hjust = 0),
        legend.position = "bottom")
```

```
# Cumulate daily intake -----
d.PrevDayFE = d.sfeed[Date == as.character('2023-07-01') & RFID %in% c('680417',
                                                                        '679459',
                                                                        '679471'),]
d.PrevDayFE[, DaySec := hour(StartTime)*3600 + minute(StartTime)*60 + second(StartTime)]

setorder(d.PrevDayFE,cols = 'RFID','DaySec')
d.PrevDayFE[, cumDMI := cumsum(IntakeKG), by = 'RFID']

p.CumIntakeDaily = d.PrevDayFE %>%
  ggplot(aes(x = as.POSIXct(StartTime), y = cumDMI,color = RFID))+
  geom_point()+
  geom_line()+
  scale_x_datetime(date_labels = "%H:%M")+
  # facet_wrap(~RFID, scales = 'free')+
  labs(x = 'Time, hour',
       y = 'Cumulative Intake, kg')+
  theme_classic()+
  theme(panel.grid.major = element_line(color = 'black', linewidth = 0.1),
        panel.grid.minor = element_line(color = 'grey', linewidth = 0.05),
        axis.text.x = element_text(angle = 45, hjust = 1),
        plot.caption = element_text(hjust = 0),
        legend.position = "bottom")
p.CumIntakeDaily
```

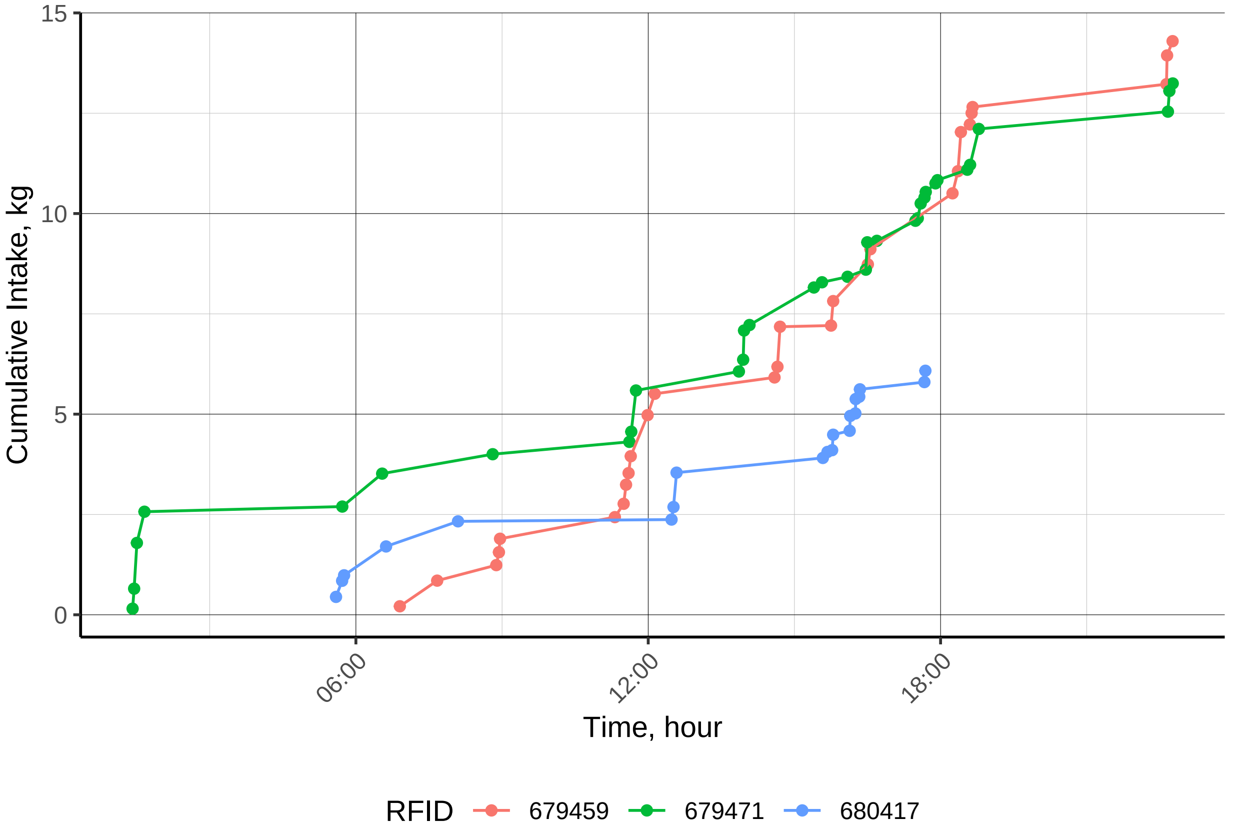
15
10
5
0
Cumulative Intake, kg
06:00
12:00
18:00
Time, hour
RFID
679459
679471
680417
