# Supplementary material for: Development of an application programming interface to automate downloading and processing of precision livestock data
Source: Transl Anim Sci. 2024 Jun 7;8:txae092. doi: 10.1093/tas/txae092 (PMC11209544; doi:10.1093/tas/txae092)

C-Lock SmartScale Tutorial

Downloading and Processing C-Lock SmartScale Data in R

Developed by Jameson Brennan, Ira Parsons, and Hector Menendez

Department of Animal Science, South Dakota State University

The objectives of this hands on tutorial are to introduce workshop participants to methods for streamlining SmartScale data processing tasks in R. This is an R Markdown document. Markdown is a simple formatting syntax for authoring HTML, PDF, and MS Word documents. For more details on using R Markdown see http://rmarkdown.rstudio.com. When you click the **Knit** button a document will be generated that includes both content as well as the output of any embedded R code chunks within the document. The example below will print a statement and run a quick computation.

Import libraries

Our first step to processing the data is to import the libraries we will use to run our analysis. Each library contains a set of functions which can be used to process data. For example, the function mean() would sum the values in a column and divide by the number of observations in the column. This code will look to see if the necessary packages are installed on your computer and if not install and load them.

```r
##if there is an error and a package or dependency needs to be updated un-comment the
#code below and replace 'rvest' with package
#remove.packages('rvest')
#install.packages('rvest')

#Needed packages
  list.of.packages <- c("rvest",'tidyverse','data.table','lubridate',
                        'knitr','markdown','fasttime','MASS')
  new.packages <- list.of.packages[!(list.of.packages %in%
                                       installed.packages()[,"Package"])]
  if(length(new.packages)) install.packages(new.packages)
library(rvest)
library(tidyverse)
library(data.table)
library(lubridate)
library(knitr)
library(rmarkdown)
library(fasttime)
library(MASS)
```

Enter your variables

The first thing you need to do is to enter your variables for the code to run. In the quotations below you will need to change the username, password, FID (or SmartScale ID), and the start and end times for the data you want to download.

Two things to note are 1) you can enter multiple greenfeeds assocated with an account and 2) the start time and end time must be in the same format. The chunk below sets the end time to the computer time for the most recent downloads.

```
#change to login user name
USERNAME <- 'demo_user'

#Change to login password
PASSWORD <- 'greenfeed'

#Change SmartScale id or id's. The second line can be uncommented out for multiple
#SmartScales notice there is no space between commas for the multiple id's
SmartScale_ID  <- '1000119'
#SmartScale_ID   <- "1000119,1000121,1000132"

#enter the start date (ST) and end date (ET) for the data you want to download
date.start <- '2023-03-14'
date.end   <- '2023-06-01'

## Data parameters -----
FULL_WEIGHT <- 1 # 1 for full body weight calculated using C-Lock algorithm, 0 for raw
#half-body weights, full weights calculated by taking the front end weight * 1.76
GET_VISITS <- 1 #Put 1 to get individual visits, Put 0 to get daily averages
```

API Code

This code chunk will pull the data from the cloud based on the specifications of what was provided above.

```
# Local system setup ----
## Choose a temp folder to download the CSV to ----
if(Sys.info()["sysname"] == "Darwin"){
  TEMP_DIRECTORY <- "/tmp" #For Mac or Linux
}else{
  TEMP_DIRECTORY <- "."    #For Windows (Maybe put C:\Users\yourusername\Desktop)
}

## Load required libraries ----
suppressWarnings(suppressMessages(try(require(rvest), silent = TRUE)))
suppressWarnings(suppressMessages(try(require(httr), silent = TRUE)))
suppressWarnings(suppressMessages(try(require(RCurl), silent = TRUE)))

## Login URL -----
login_url <- "https://greenfeed.c-lockinc.com/GreenFeed/checklogon.php"

## API URL -----
if (GET_VISITS == GET_VISITS) {
  #Download URL - Individual Visits Weights
  download_url <- paste0("https://greenfeed.c-lockinc.com/GreenFeed/tabledata/sfanimals/getanimalweight
                         "&fids=0,", SmartScale_ID,
                         "&st=", date.start,
                         "&et=", date.end,
                         "&full=", FULL_WEIGHT);
} else {
```

```
  #Download URL - Daily Average Weights
  download_url <- paste0("https://greenfeed.c-lockinc.com/GreenFeed/tabledata/sfanimals/animalweights.pl
                        "&fids=0,", SmartScale_ID,
                        "&from=", date.start0,
                        "&to=", date.end,
                        "&full=", FULL_WEIGHT);
}

# Download data ----
## Log into the website using URL requests -----
{
  #Create session then download form data
  session <- session("https://greenfeed.c-lockinc.com/GreenFeed")
  form <- html_form(read_html(login_url))[[1]]

  #Set login credentials
  form <- set_values(form, username = USERNAME)
  form <- set_values(form, password = PASSWORD)
  suppressWarnings(form <- set_values(form, redir = "home.php?logout")) #This will give you a warning -

  #Save main page url
  suppressMessages(main_page <- submit_form(session, form))

  #Download the data
  download <- jump_to(main_page, download_url)
}

# Decode binary data ----
#Because the downloaded data is binary data, you must write it to a temporary file then read it back as
## Write data to a file ----
FILENAME <- paste0(TEMP_DIRECTORY, "/C.txt")
writeBin(download$response$content, FILENAME)

#Read the CSV into a df dataframe
if (GET_VISITS == 1) {
  #SmartScale Headers are: "Link" "StartTime" "StopTime" "FeederID" "Duration" "AnimalName" "RFIDTag" "
  colclasses=c("character", "POSIXct", "POSIXct", "integer", "integer", "character", "character", "inte
} else {
  #SmartScale Headers are: "Link" "StartTime" "StopTime" "FeederID" "Duration" "AnimalName" "RFIDTag" "
  colclasses=c("character", "character");
}

d.smart = fread(file = FILENAME, header=TRUE, sep=",", quote="\"", colClasses = colclasses) # Read into
d.smart[d.smart == 0] <- NA # Replace 0's with NA
```

Viewing and cleaning SmartScale data

This is the raw dataset downloaded from the SmartScale API.The code chunk will print the first five rows of data in table format.

```
knitr::kable( head(d.smart))
```

View	StartTime	StopTime	FeederID	Duration	AnimalName	RFIDTag	Weight	Valid
https://greenfeed.c-lockinc.com/GreenFeed/sfdata.php?fid=1000119&dt=2023-03-14%2018:47:22&dur=60&s1=20&s2=23	2023-03-14 18:47:32	2023-03-14 18:48:05	1000119	33	SHSF K30	000000000840003241679559	559	
https://greenfeed.c-lockinc.com/GreenFeed/sfdata.php?fid=1000119&dt=2023-03-14%2019:30:08&dur=60&s1=20&s2=23	2023-03-14 19:30:18	2023-03-14 19:30:34	1000119	16	SHSF K41	000000000840003241679563	490	
https://greenfeed.c-lockinc.com/GreenFeed/sfdata.php?fid=1000119&dt=2023-03-14%2019:30:27&dur=60&s1=20&s2=23	2023-03-14 19:30:37	2023-03-14 19:30:55	1000119	18	SHSF K41	000000000840003241679563	486	
https://greenfeed.c-lockinc.com/GreenFeed/sfdata.php?fid=1000119&dt=2023-03-14%2019:30:57&dur=60&s1=20&s2=23	2023-03-14 19:31:07	2023-03-14 19:31:20	1000119	13	SHSF K41	000000000840003241679563	301	N
https://greenfeed.c-lockinc.com/GreenFeed/sfdata.php?fid=1000119&dt=2023-03-14%2020:01:56&dur=60&s1=20&s2=23	2023-03-14 20:02:06	2023-03-14 20:02:22	1000119	16	SHSF K41	000000000840003241679563	495	
https://greenfeed.c-lockinc.com/GreenFeed/sfdata.php?fid=1000119&dt=2023-03-14%2020:08:08&dur=60&s1=20&s2=23	2023-03-14 20:08:18	2023-03-14 20:08:30	1000119	12	Beach K224	000000000840003241679593	350	N

We can see that the 'view' column is likely not necessary and can be removed. In addition, we will create a new column called 'Date' that converts the start time to a Date only value. Lastly we will convert the 14 digit RFID number to only the last 6 digits to simplify identifying unique animals.

```
#Remove unnecessary columns
d.smart$View=NULL


#convert date time to date value to look at daily visits
d.smart$Date=as.Date(d.smart$StartTime)
d.smart$RFIDTag= stringr:: str_sub(d.smart$RFIDTag,-6,-1) #get last 6 digits of RFID
```

One of the first steps to processing and cleaning data is to plot it. This code chunk will get the number of daily visits for each scale and plot it by day.

Quick Visualization Plots

This next section is used to generate a series of quick plots to summarize visits and data from the SmartScales to check usage rates. This can be helpful to plot is animals are routinely using the equipment and to see if there are any sudden changes in usage rate that may indicate either a issue with the technology or access.

```
#get the number of unique animals visiting by day
rfid_day=d.smart %>%                              # Applying group_by & summarise
  group_by(Date,FeederID) %>%
  summarise(count = n_distinct(RFIDTag))
```

```
rfid_day=na.omit(rfid_day)

ggplot(rfid_day,aes(x=Date,y=count))+
  geom_bar(stat = 'identity')+
  facet_wrap(~FeederID)+
  ggtitle('Number of Unique animals per day')
```

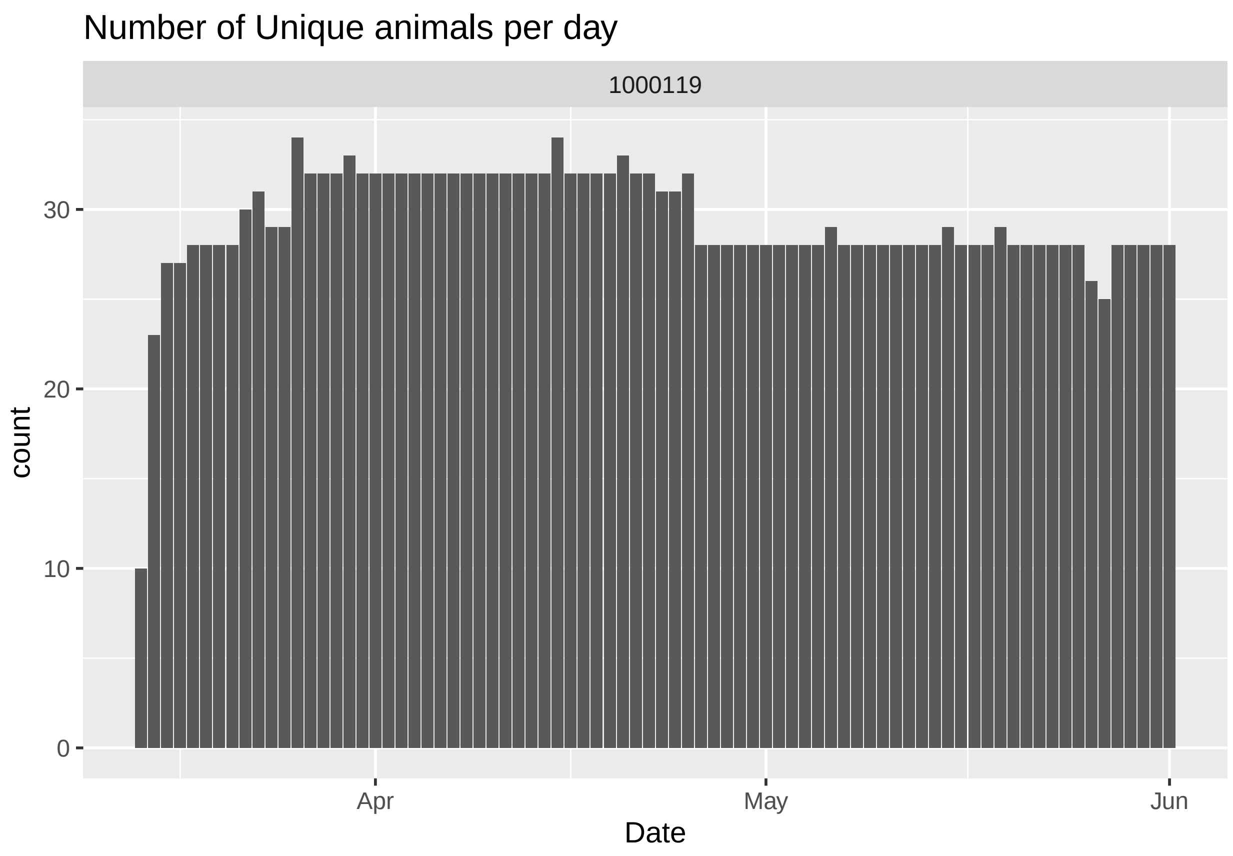


```
#convert start time to date
d.smart$Date=as.Date(d.smart$StartTime)

d.smart$RFID=stringr:: str_sub(d.smart$RFID,-6,-1)

#get a count on the number of good observations by each greenfeed by day
library(dplyr)
daily_good_data=d.smart %>% count(FeederID, Date, sort = TRUE)

daily_good_data=na.omit(daily_good_data)
ggplot(daily_good_data,aes(x=Date,y=n))+
  geom_bar(stat = 'identity')+
  facet_wrap(~FeederID)+
  ggtitle('Number of observations by SmartScale by day')+
  ylab('Number of daily observations')
```

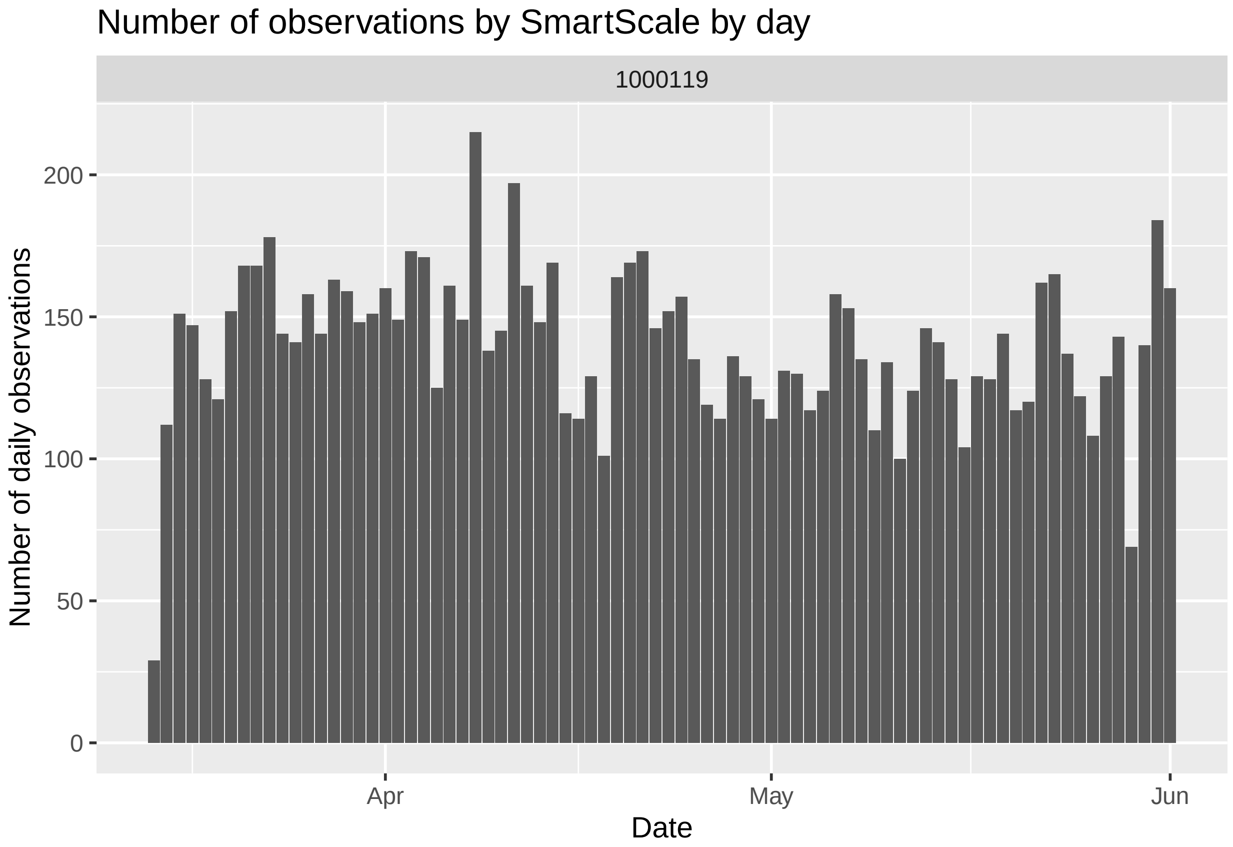

The smartscale data has a column named 'Valid' that flags potentially bad data in the system based on quantiles. The following plots show the bad data labeled as Valid = No for the entire dataset and for an individual animal.

```
#plot animal weight by date
ggplot(d.smart,aes(x=Date,y=Weight,color=Valid))+
  geom_point()
```

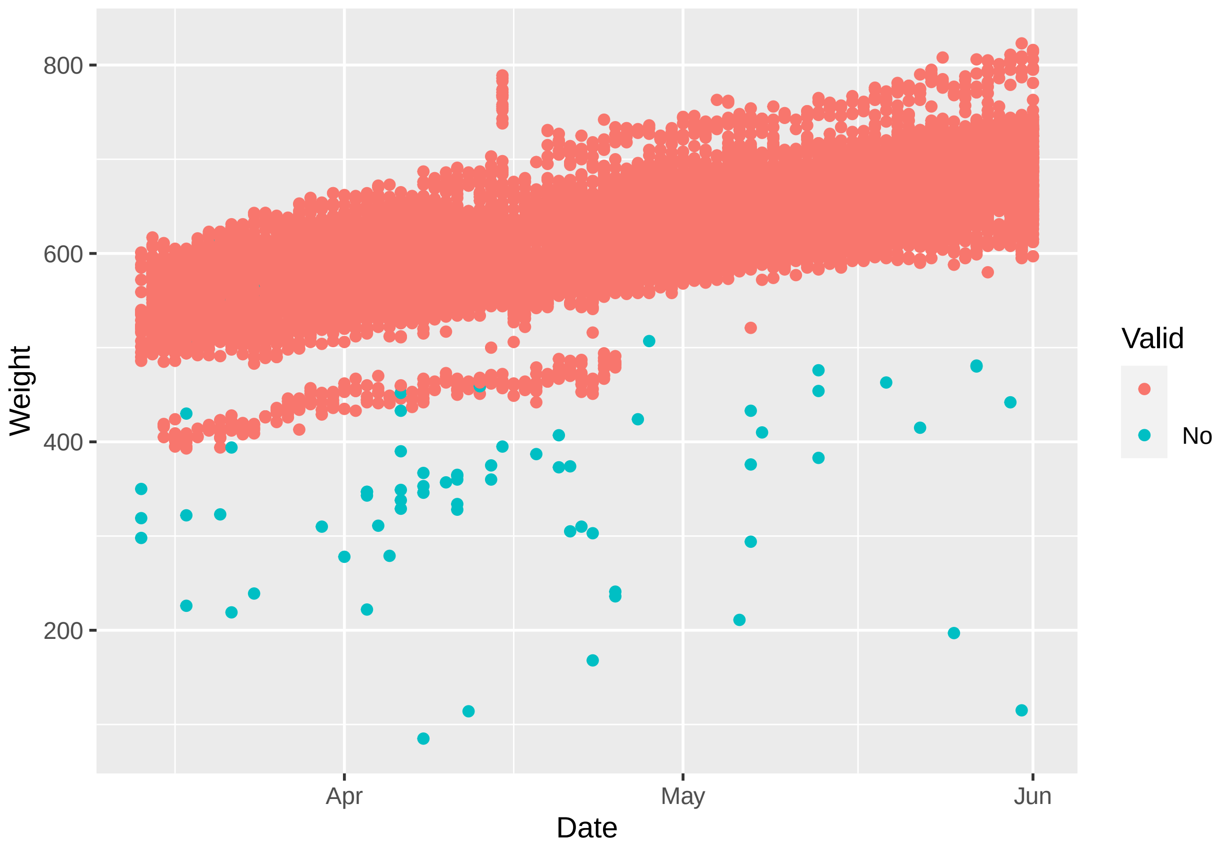


```
ggplot(subset(d.smart,RFIDTag=='679563'),aes(x=Date,y=Weight,color=Valid))+
  geom_point()
```

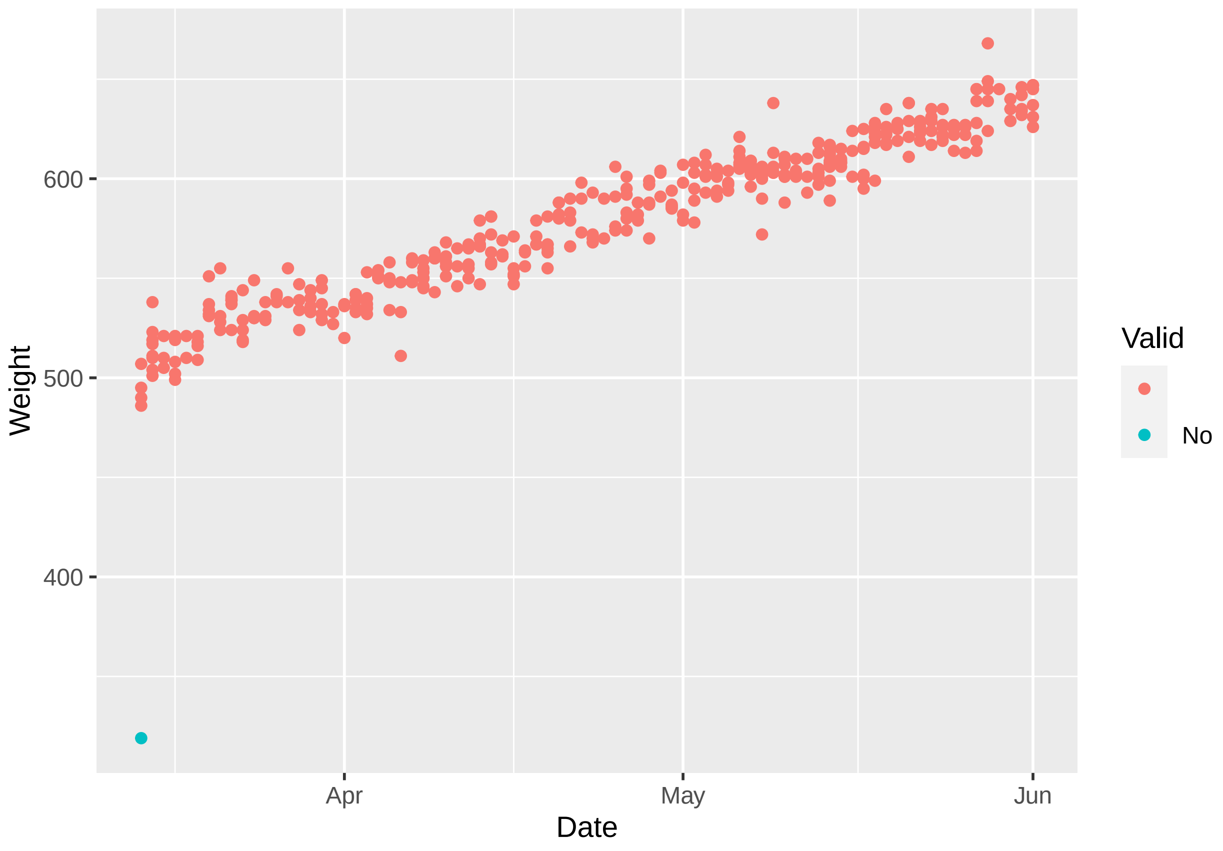

This next chunk of code will remove the observations that are not valid and replot the data.

```
#Remove not valid points
d.smart=subset(d.smart,Valid!='No')
#plot animal weight by date
ggplot(d.smart,aes(x=Date,y=Weight))+
  geom_point()
```

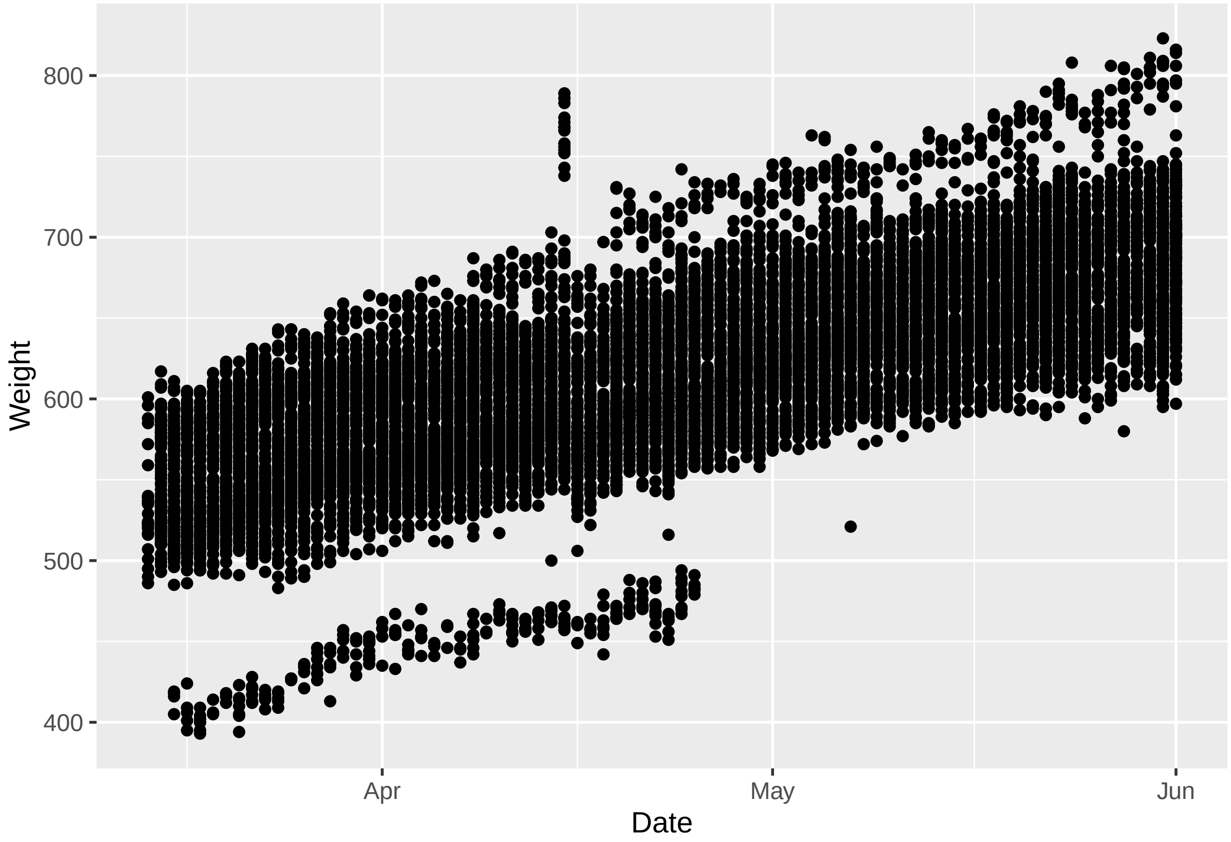

Further Data Cleaning and Filtering

This next code chunk will add day of trial to the dataframe which will help for calculating average daily gain (ADG)

```
# Add days to data
d.tdays = data.table(Date = seq.Date(from = as.Date(min(d.smart$Date)),
                                     to = as.Date(max(d.smart$Date)), by = 1),
                     tday = as.numeric(seq(from = 0,
                                           to = difftime(max(d.smart$Date), min(d.smart$Date), units =
d.smart = d.tdays[d.smart, on = 'Date']
d.smart[, Weight:= as.numeric(Weight)]
d.smart[, RFIDTag := as.character(RFIDTag)]
```

In addition to flagging the data as valid or not valid, Parsons et al., 2023 proposed an additional method using robust regression to remove potentially bad data from in pasture weighing systems. We will apply that method below.

```
# Filter Spurious weights using robust regression (Parsons et al., 2023)
m.rob = rlm(Weight ~ RFIDTag + tday, data = d.smart)
d.smrtrob = data.table(FeederID = d.smart$FeederID,
                       RFIDTag = d.smart$RFIDTag,
                       Date = d.smart$Date,
                       tday = d.smart$tday,
```

```
                       StartTime = d.smart$StartTime,
                       Duration = d.smart$Duration,
                       Weight = d.smart$Weight,
                       resid = m.rob$residuals,
                       hwt = m.rob$w)

## Assign outliers
d.smrtrob[, Outlier := fifelse(hwt > 0.99, 'In Range','Outlier')]

ggplot(subset(d.smrtrob,RFIDTag=='679563'),aes(x=Date,y=Weight,color=Outlier))+
  geom_point()
```

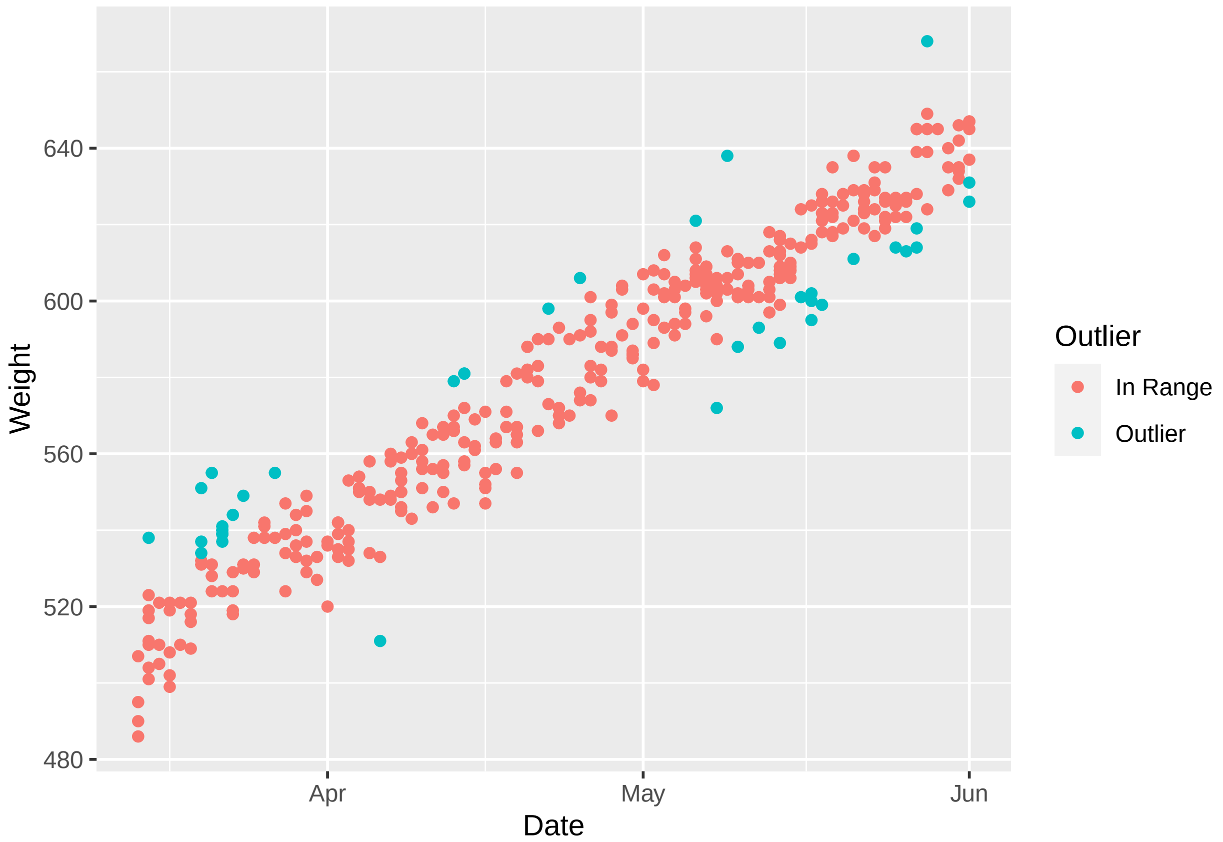


```
d.smart = d.smrtrob[Outlier == 'In Range', ] # Filter to in range points
```

Using the table function we will get the number of daily weights for each individual animal. We can see that several tags only have a few observations while others have hundreds. These are could be test tags that were used to test the scales or had animals that infrequently used the equipement. The code below will delete individual tags with less than 6 observations.

```
table(d.smart$RFIDTag)
```

```
##
## 030197 030226 030296 030312 605891 605892 605893 605896 605899 679558 679559
##      6      1      2      1    242    252    368    355    325    282    241
```

```
## 679560 679563 679572 679573 679590 679591 679593 679594 679596 679605 679608
##    243    337     70     57    402    231    229    341    317    132    337
## 679612 679617 679619 679622 679629 679633 679635 679663 679795 679814 679817
##    143    337    337    212    175    240    293      2      4      2      6
## 679822 680396 680410 680411 680412 680413 680414 828028 828031 828038
##      2    245    260    336    259    270    306    218    296    339
```

```
d.smart=d.smart %>% group_by(RFIDTag) %>% filter(n()>= 6) %>% ungroup()
table(d.smart$RFIDTag)
```

```
##
## 030197 605891 605892 605893 605896 605899 679558 679559 679560 679563 679572
##      6    242    252    368    355    325    282    241    243    337     70
## 679573 679590 679591 679593 679594 679596 679605 679608 679612 679617 679619
##     57    402    231    229    341    317    132    337    143    337    337
## 679622 679629 679633 679635 679817 680396 680410 680411 680412 680413 680414
##    212    175    240    293      6    245    260    336    259    270    306
## 828028 828031 828038
##    218    296    339
```

Calculate Average Daily Gain

Next we want to calculate the average daily gain. To do so we can fit a linear regression to get a model for to estimate weight by day of trial. If we wanted to fit a linear model to the entire herd we can do so using all available data with Weight as our y and trial day as our x. The code below fits this linear model.

```
model_all_animals=lm(Weight~tday,data=d.smart)
summary(model_all_animals)
```

```
##
## Call:
## lm(formula = Weight ~ tday, data = d.smart)
##
## Residuals:
##      Min       1Q   Median       3Q      Max
## -152.417  -23.256   -3.834   28.501  171.551
##
## Coefficients:
##              Estimate Std. Error t value Pr(>|t|)
## (Intercept) 536.47638    0.85231   629.4   <2e-16 ***
## tday          1.96788    0.01905   103.3   <2e-16 ***
## ---
## Signif. codes:  0 '***' 0.001 '**' 0.01 '*' 0.05 '.' 0.1 ' ' 1
##
## Residual standard error: 39.93 on 9037 degrees of freedom
## Multiple R-squared:  0.5414, Adjusted R-squared:  0.5413
## F-statistic: 1.067e+04 on 1 and 9037 DF,  p-value: < 2.2e-16
```

We can see that the intercept of the model is 536, which would equate to our average starting weight for the herd and the slope is 1.96 which would equate to our average daily gain (ADG). Plotting it we can see the relationship.

```
ggplot(d.smart,aes(y=Weight,x=tday))+
  geom_point()+
  geom_smooth(method=lm)
```

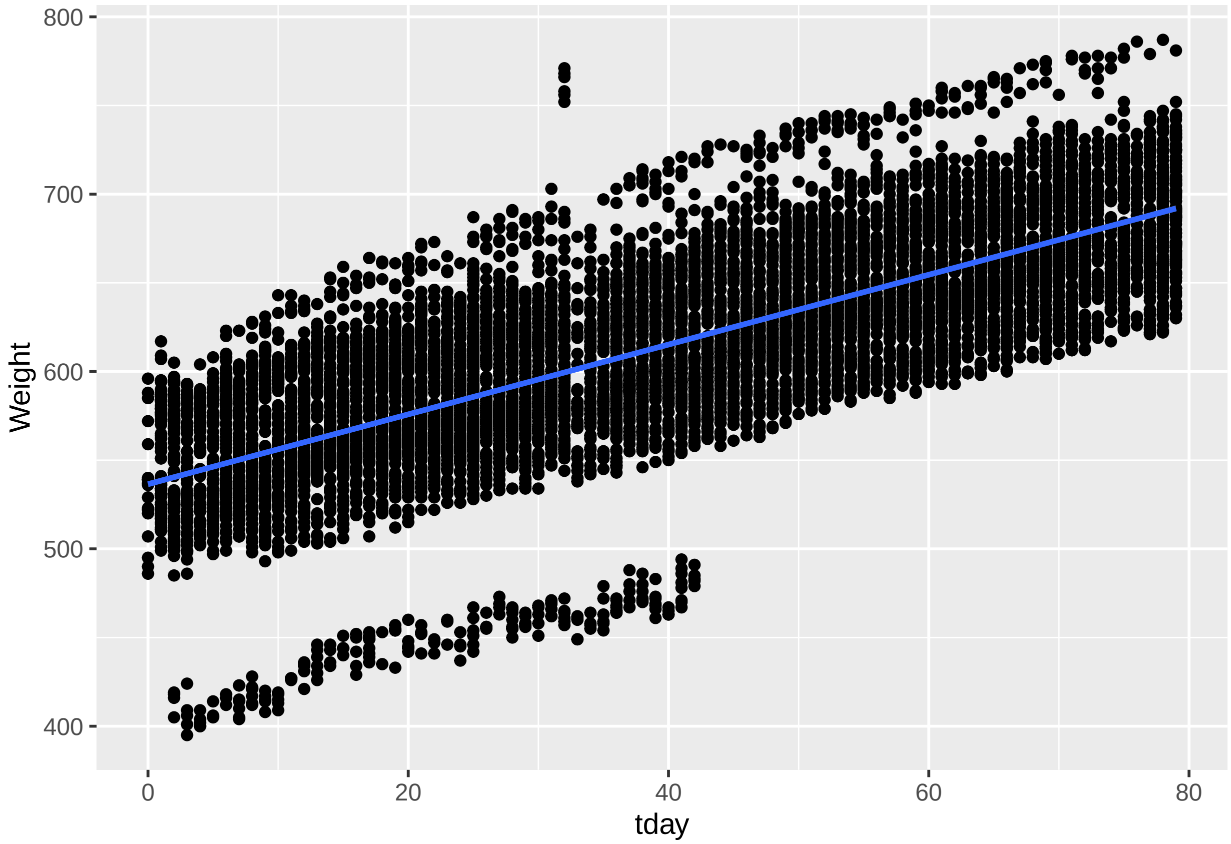

Though herd level ADG is important, using precision data we can also look at individual animal ADG. To do so we need to run a linear model for each individual animal. The code below will loop through each individual animal, subset the data based on RFID tag, create a linear model as above, extract the slope and intercept for the model, and save them in a new dataframe that contains the RFIDtag, slope, and intercept for each individual animal.

```
ADG_individual=data.frame()


for (i in 1:length(unique(d.smart$RFIDTag))){

    sublm=subset(d.smart,RFIDTag==unique(d.smart$RFIDTag)[i])
    mod=lm(sublm$Weight~sublm$tday)
    int= mod$coefficients[1]
    slope=mod$coefficients[2]


    sub_df=data.frame(unique(sublm$RFIDTag),int,slope)
    ADG_individual=rbind(ADG_individual,sub_df)
}

row.names(ADG_individual) <- NULL
```

Plotting the data we can see the slope and intercept for each animal with the RFID tag.

```r
colnames(ADG_individual)=c('RFIDTag','Intercept','Slope')


ggplot(ADG_individual,aes(x=Intercept,y=Slope,label=RFIDTag))+
  geom_point(size=2,color='Red')+
  geom_text(hjust=0, vjust=0)+
  ggtitle('ADG and Starting Weight for Individual Animals')+
  xlab("\nStarting Weight (Intercept) in Lbs")+
  ylab('ADG (Slope) in Lbs \n')
```

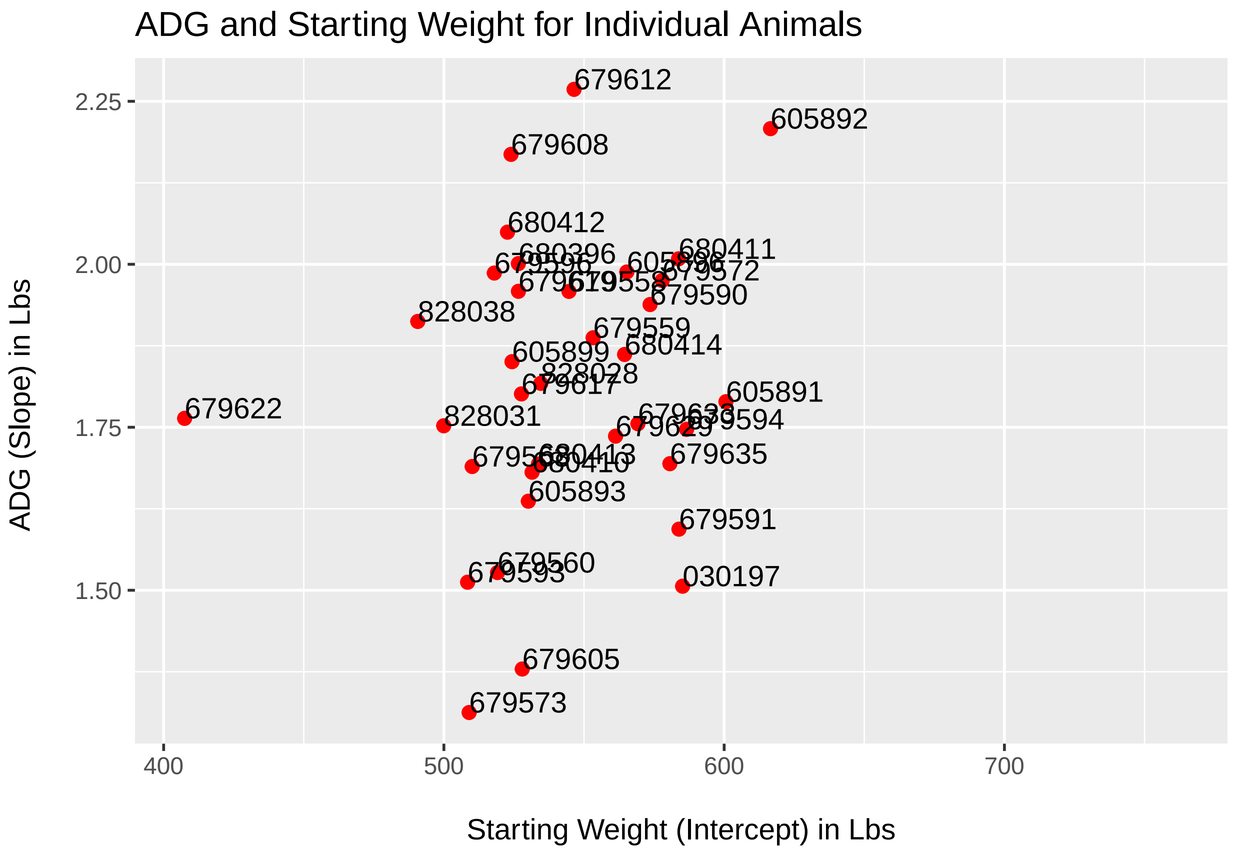

Supplement: txae092_suppl_Supplementary_Material [file txae092_suppl_supplementary_material.zip › SmartScale_Markdown.pdf]
